# A de novo silencer causes elimination of *MITF-M* expression and profound hearing loss in pigs

**DOI:** 10.1186/s12915-016-0273-2

**Published:** 2016-06-27

**Authors:** Lei Chen, Weiwei Guo, Lili Ren, Mingyao Yang, Yaofeng Zhao, Zongyi Guo, Haijin Yi, Mingzhou Li, Yiqing Hu, Xi Long, Boyuan Sun, Jinxiu Li, Suoqiang Zhai, Tinghuan Zhang, Shilin Tian, Qingyong Meng, Ning Yu, Dan Zhu, Guoqing Tang, Qianzi Tang, Liming Ren, Ke Liu, Shihua Zhang, Tiandong Che, Zhengquan Yu, Nan Wu, Lan Jing, Ran Zhang, Tao Cong, Siqing Chen, Yiqiang Zhao, Yue Zhang, Xiaoqing Bai, Ying Guo, Lidong Zhao, Fengming Zhang, Hui Zhao, Liang Zhang, Zhaohui Hou, Jiugang Zhao, Jianan Li, Lijuan Zhang, Wei Sun, Xiangang Zou, Tao Wang, Liangpeng Ge, Zuohua Liu, Xiaoxiang Hu, Jingyong Wang, Shiming Yang, Ning Li

**Affiliations:** State Key Laboratory for Agrobiotechnology, College of Biological Sciences, National Engineering Laboratory for Animal Breeding, China Agricultural University, Beijing, 100193 China; Department of Otolaryngology, Head & Neck Surgery, Institute of Otolaryngology, Chinese PLA General Hospital, Beijing, 100853 China; Key Laboratory of Pig Industry Sciences (Ministry of Agriculture), Chongqing Academy of Animal Science, Chongqing, 402460 China; Institute of Animal Genetics and Breeding, College of Animal Science and Technology, Sichuan Agricultural University, Ya’an, Sichuan 625014 China; Department of Communicative Disorders and Sciences, Center for Hearing and Deafness, State University of New York at Buffalo, Buffalo, New York USA

**Keywords:** De novo silencer, *MITF-M*, Hearing loss, Waardenburg syndrome, *cis-*regulatory element, Pig

## Abstract

**Background:**

Genesis of novel gene regulatory modules is largely responsible for morphological and functional evolution. De novo generation of novel *cis*-regulatory elements (CREs) is much rarer than genomic events that alter existing CREs such as transposition, promoter switching or co-option. Only one case of de novo generation has been reported to date, in fish and without involvement of phenotype alteration. Yet, this event likely occurs in other animals and helps drive genetic/phenotypic variation.

**Results:**

Using a porcine model of spontaneous hearing loss not previously characterized we performed gene mapping and mutation screening to determine the genetic foundation of the phenotype. We identified a mutation in the non-regulatory region of the melanocyte-specific promoter of microphthalmia-associated transcription factor (*MITF*) gene that generated a novel silencer. The consequent elimination of expression of the *MITF-M* isoform led to early degeneration of the intermediate cells of the cochlear stria vascularis and profound hearing loss, as well as depigmentation, all of which resemble the typical phenotype of Waardenburg syndrome in humans. The mutation exclusively affected *MITF-M* and no other isoforms. The essential function of *Mitf-m* in hearing development was further validated using a knock-out mouse model.

**Conclusions:**

Elimination of the *MITF-M* isoform alone is sufficient to cause deafness and depigmentation. To our knowledge, this study provides the first evidence of a de novo CRE in mammals that produces a systemic functional effect.

**Electronic supplementary material:**

The online version of this article (doi:10.1186/s12915-016-0273-2) contains supplementary material, which is available to authorized users.

## Background

Genome variation in non-coding regions may not only disrupt regulatory elements but can also create them [1]. Due to the sequence-specific nature of transcription factors’ binding to DNA, introduction of a single-nucleotide polymorphism (SNP) or a small-sized insertion or deletion (indel) in the target regulatory sequence can incapacitate or inhibit binding [1, 2]. Moreover, these relatively small mutations can be sufficient to generate a novel *cis*-regulatory element (CRE). A growing body of evidence has shown that new regulatory behavior can accompany the modification of existing functional elements [3–5], with point mutations and deletions being sufficient to generate *cis*-regulatory divergences [6, 7].

One of the most well studied genetic events capable of creating new regulatory behaviors is the transposon, a transposable genetic element that has been shown to generate functional changes in existing enhancers; the extensive studies on transposons have revealed their activities in insects and plants as well as mammals [8, 9]. In contrast, de novo generation of a novel CRE is less well studied. Only one case has been reported to date, that of an enhancer generated by whole-genome duplication in sequences in fish that were demonstrated as formerly lacking *cis*-regulatory activity [10]. No such cases have been reported for mammals, and there are no reports of minor changes driving either de novo genesis of regulatory elements or systemic functional alterations.

Waardenburg syndrome type 2 (WS2; OMIM #193510) is a hereditary sensorineural deafness syndrome caused by gene mutations; additional physical traits of the disease in humans include heterochromia iridis and white forelock [11, 12]. A common mutation found in WS2 sufferers involves the microphthalmia-associated transcription factor (*MITF*), which plays a critical role in melanocytes and melanoma [13, 14]. The *Mitf* multi-promoter gene encodes at least seven isoforms of *MITF*, each with a distinct N-termini. These seven isoforms have been identified in humans and mice, and are known to be translated from different transcriptional variants with differing first and/or second 5’-end exons [15]. The various promoters associated with each isoform contribute to their tissue-specific expression and functions [16].

**Fig. 1:**
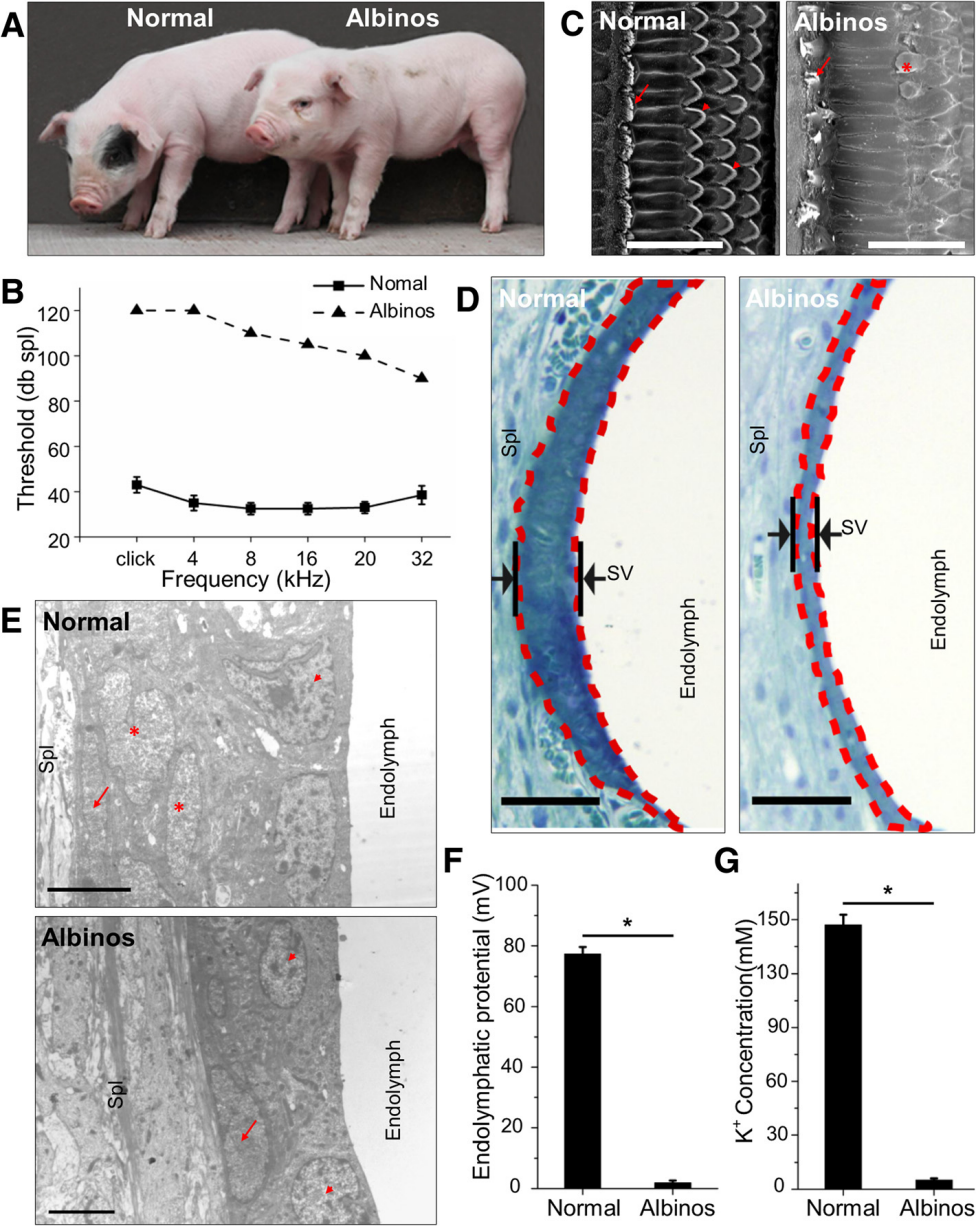
Cochlear morphology and auditory electrophysiology defects of albino pigs. **a** Gross image of a normal pig and an albino pig. **b** Results of auditory brainstem response tests showing profound hearing loss of albino pigs. The raw data is provided in Additional file 5: sheet 1 Data of ABR tests (pigs). **c** Scanning electron microscopy images showing missing or fused (star) stereocilias of inner (arrow) and outer (arrowhead) hair cells in albino pigs. **d** Images showing that the stria vascularis (SV) of albino pig are remarkably thinner than that of normal pig. **e** Image showing lack of intermediate cells in the SV of albino pigs. Marginal cell layer, arrowheads; intermediate cell, stars; basal cell, arrows; spiral ligament, Spl. **f** and **g** The average values of endolymphatic potential and scala media potassium concentration in albino pigs were significantly lower than in normal pigs (raw data in Additional file 5: sheet 2 Data of EP and sheet 3 Data of K+ concentration). Error bars indicate the standard deviations. Scale bars in **c** = 100 μm, in **d** = 50 μm, and in **e** = 5 μm

In the present study, we described a porcine model with spontaneous deafness, which exhibits WS2-like phenotypes, including depigmentation (Fig. 1). We performed whole-genome mapping and detected a short insertion in the distal melanocyte-specific regulatory region of *MITF*. We showed that this insertion creates a de novo silencer that completely eliminated the expression of the transcripts for the *MITF-M* isoform both in vivo and in vitro. Therefore, the present study provides the first evidence to demonstrate that minor mutations in non-coding regions that lack *cis*-regulatory activity are able to generate a systemic, functional de novo silencer and result in dramatic phenotypic alterations. Additionally, we determined that among all of the *MITF* isoforms only *MITF-M* was affected in this porcine model and confirmed the phenotype relationship using an M-exon knock-out mouse model; thus, we conclude that *MITF-M* is vital for normal hearing and may play an important role in WS2 of mammals, including humans.

## Results

### Profound hearing loss in a porcine model

Albino pigs (Fig. 1a) spontaneously arising from a native breed of swine in Southwest China (Rongchang pigs [17]) are well-studied for their observed phenotypes of deafness and depigmentation, similar to the phenotype of WS2 [11, 12]. Results from auditory brainstem response (ABR) tests show that the albino pigs produced no recognizable waveforms up to 100 dB sound pressure level (SPL) stimuli in the range from 4–32 kHz, whereas normal pigs produced ABR thresholds at 5–10 dB SPL (Fig. 1b). Loss of hair cells and stereocilia bundles were observed in the cochleae of the albino pigs by scanning electron microscopy (SEM; Fig. 1c). Because the hearing loss observed in human cases of WS2 is attributed to abnormal cochlear stria vascularis (SV) [18], the morphology of SV was examined in our study’s albino pigs using light microscopy and transmission electron microscopy (TEM). As shown in Fig. 1d and e, the albino pigs lacked intermediate cells and had thinner SVs, consisting of two layers of cells only. Because the major functions of the SV are secretion of potassium ions and production of endolymphatic potential (EP), we recorded EPs and measured the [K^+^] in the scala media of the cochlea. The EP and [K^+^] were significantly lower than those of normal pigs (*P* < 0.001, Student’s *t*-test; Fig. 1f, g). Since EP and high [K^+^] in the endolymph are reportedly the driving force for mechanotransduction in cochlear hair cells [19], a reduction in EP can lead to profound hearing loss. All these phenotypes were tested at postnatal day 13. Thus, these results confirmed the phenotype of profound hearing loss related to cochlear morphology defects in our study’s albino pigs. Considering that eye defects have also been observed in some WS2 patients, we also assessed the morphology of porcine eyes. The irises of the albino pigs presented with pale coloration due to lack of pigmentation (Additional file 1: Figure S1A). The paraffin-embedded sections of retinae showed hypopigmentation in the choroid, but the retinal pigment epithelium was normal in both the normal and albino pigs (Additional file 1: Figure S1B).

### Gene mapping and mutation screening

**Fig. 2:**
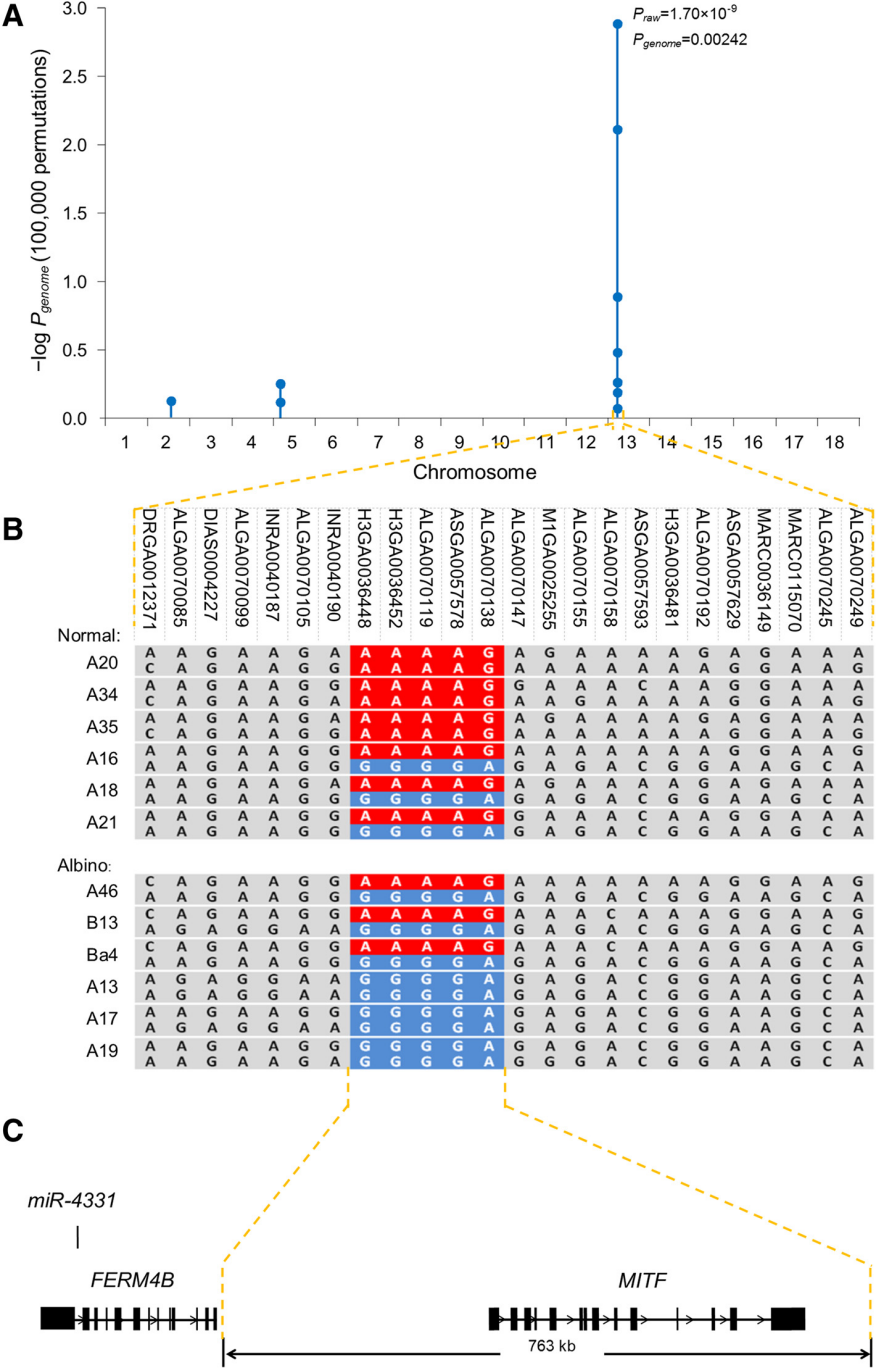
Genome-wide association mapping of the hearing loss trait in albino pigs. **a** The strongest association was identified on chromosome 13 by case-control association (*P*_genome_ = 0.00242) using the whole-genome data analysis toolset PLINK. **b** Haplotype sharing analysis showed a perfect concordance of a haplotype with hearing loss phenotype in the mapped population. A 767-kb associated interval (from INRA0040190 to ALGA0070147) was defined by five single nucleotide polymorphism markers in that haplotype and proximal recombinant markers. **c** *Mitf* is the only known gene located in that region

To investigate whether the hearing loss in our study’s albino pigs was caused by genetic factors, a genetic analysis was performed with the aim of identifying the hereditary pattern of the deafness. The deafness incidence rate in offspring of albino × albino mating was 100 %, whereas almost all offspring of normal × albino mating yielded normal hearing offspring. Moreover, there was no significant difference in the prevalence of deafness between males and females. These results implied an autosomal recessive inheritance pattern; to confirm this, 11 pairs of putative heterozygous boars and sows were selected from the normal herd according to the selection criteria of having produced at least one albino offspring. In total, 74 piglets from 11 litters of heterozygous × heterozygous matings were phenotyped to investigate the Mendelian segregation ratio; the results were 25 piglets with hearing loss and 49 piglets with normal hearing. Results of χ^2^ goodness-of-fit test indicated that the segregating ratio of the hearing loss trait was 3:1 (*P* < 0.01; Additional file 2: Table S1), confirming that the trait’s inheritance mode was autosomal recessive. This result led us to hypothesize that all of the albino pigs had inherited a mutant allele (r allele) from a common ancestor, instead of the wild-type allele (R allele) of normal pigs. We then applied a whole genome association approach with a phenotype-segregated population (Additional file 1: Figure S2). The strongest association signals were detected at two markers (ASGA0057578 and ALGA0070138, *P*_genome_ = 0.00242; Fig. 2a) on *Sus scrofa* chromosome 13 (SSC 13). Then, by using haplotype association analysis we detected strong concordance of a haplotype block that was composed of five markers, with the hearing loss phenotype in the mapping population (Fig. 2b). All homozygotes of the “GGGGA” haplotype were hearing impaired and the homozygotes of the “AAAAG” haplotype had normal hearing (*P*_raw_ = 3.72 × 10^–12^; chromosome-wide significance, *P*_chr_ = 1.74 × 10^–5^, 25,000 permutations; Fig. 2b). Among the heterozygotes, a small percentage of the pigs (4/51) were hearing impaired, indicating an autosomal semi-recessive transmission mode for hearing loss. Based on these results, we mapped the causative mutant gene of hearing loss to a 763 kb interval (SSC 13: 56,170,062 to 56,933,573), which was defined by the haplotype block and the proximal recombinant markers (Fig. 2c). The melanogenesis- and hearing-related gene *Mitf* was the only annotated gene located in this sequence interval (Fig. 2c). A search of the literature determined that previous studies had associated mutations in *Mitf* with auditory-pigmentary syndrome in humans, mice, cattle, horses and dogs [20–24], all of which have reported phenotypes similar to those of the albino pigs used in our study. Based on these results we speculated that *Mitf* was likely the causative mutant gene of hearing loss in these albino pigs.

To achieve more fine mapping of the causative mutation, we carried out gene screening for 12 mutant (*Mitf* ^*r/r*^) and 12 wild-type (*Mitf*^*R/R*^) pigs. The porcine *Mitf* gene was not completely annotated in the reference genome (*Sus scrofa* genome 10.2) due to the poor assembly quality in this region. Therefore, we performed a homology annotation using the human *Mitf* mRNA from the Reference Sequence (RefSeq) database and the scaffold (TP_scaffold_24421) from our recently reported genome of Tibetan wild boars [25]. We found that 15 exons, spanning 243 kb of consecutive sequence, had a high syntenic relationship with the *Mitf* genes in both human and mouse (Additional file 2: Table S2). We next amplified and sequenced all exons, exon-intron boundaries and proximal promoters of the full *Mitf* gene, out to 10 kb upstream of the transcription start site of the encoded *MITF-M* isoform. In total, 21 co-segregated variants were identified, including 16 SNPs, four insertions, and one deletion (Additional file 2: Table S3). A C > A non-synonymous variant, which induced a N106K transition, was detected in the protein-coding region (exon 3). However, because only the *Mitf* ^*R/R*^ pigs carried this variant, it seemed unlikely to be correlated with the hearing loss phenotype. The other 20 variants were located in regulatory regions.

As the associated region we discovered was too large for complete screening, the causative mutation could have been missed due to incomplete coverage by Sanger sequencing. To help rule out this possibility, we used whole-genome re-sequencing data for three of the *Mitf* ^*r/r*^ pigs and three of the *Mitf* ^*R/R*^ pigs (Additional file 2: Table S4). A total of 1711 SNPs were detected in the associated region (SSC13: 56,170,062 to 56,933,573; Additional file 3: Table S5) and 961 of these co-segregated with the hearing loss phenotype, including 362 ambiguous SNPs with missing data (Additional file 3: Table S6). Furthermore, 103 open-access porcine whole genome re-sequencing data sets (Additional file 2: Table S4) were included in the analysis to filter out common variants, which are not expected to be related to the hearing loss phenotype. Because this phenotype has not been reported in any other pig breed beyond the Rongchang breed, the 103 pigs from the open-access database were regarded as wild-type pigs (*MITF* ^*R/R*^). Next, 946 of the abovementioned 961 co-segregated SNPs were sought in the 103 *MITF* ^*R/R*^ pigs (Additional file 3: Table S6); only 15 of the co-segregated SNPs were identified as carried exclusively by the *Mitf* ^*r/r*^ pigs (Additional file 3: Table S7). Additionally, nine of those 21 co-segregated variants detected in the mutation screening noted above were excluded due to their existence in any *Mitf* ^*R/*R^ pigs (marked with bold text in Additional file 2: Table S3). Combining the data from our mutation screening and re-sequencing analysis provided a total of 26 co-segregated variants that were deemed as candidate mutations for further study (Additional file 4: Table S8).

### *Mitf* expression analysis

**Fig. 3:**
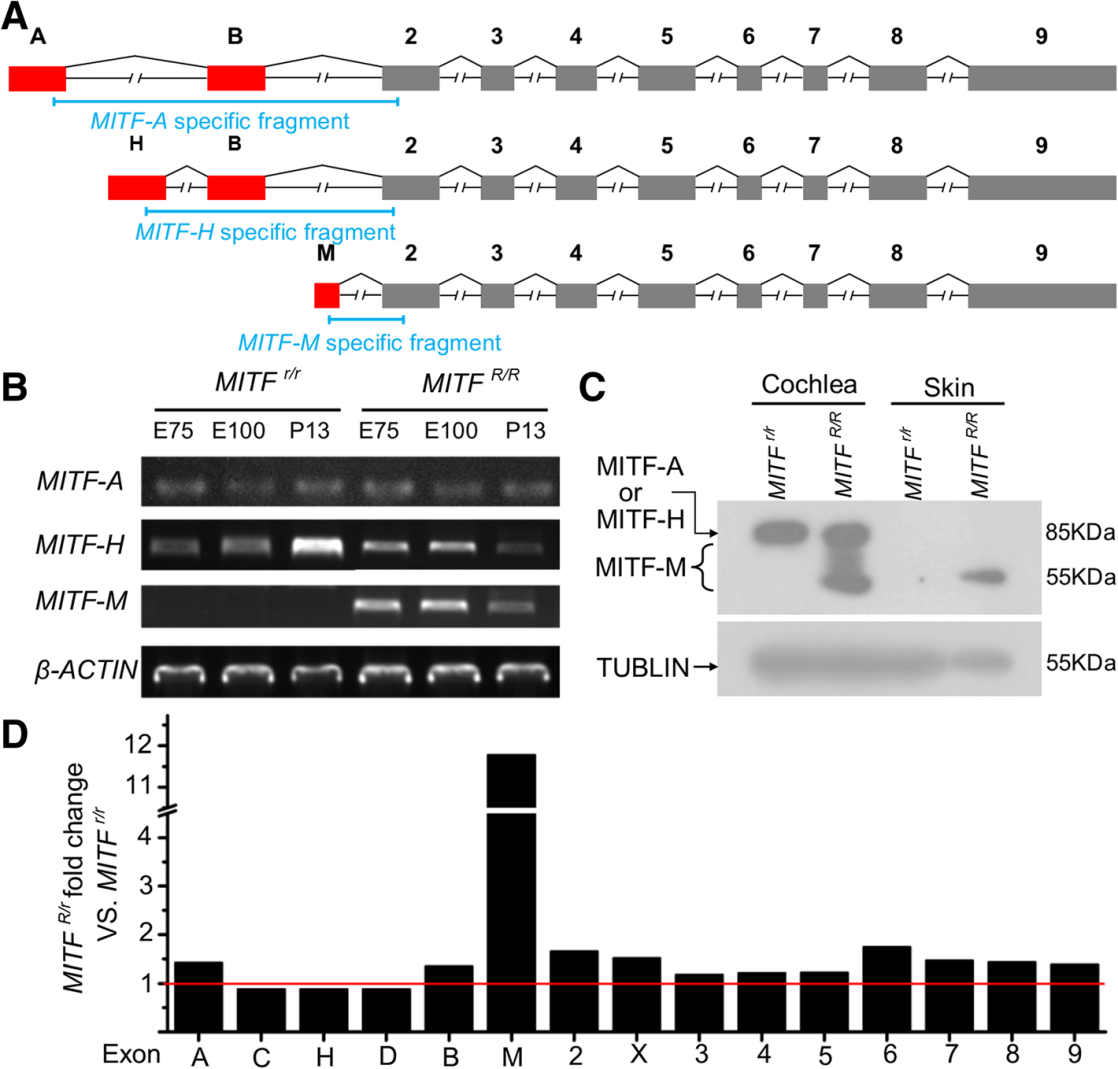
Expression analysis of *Mitf* transcriptional variants and isoforms. **a** Schematics of splicing structure in the porcine *Mitf* transcript variants (leading to *MITF-A*, *MITF-H* and *MITF-M*) detected in the cochlea. The specific fragments of transcript variants used for quantitative PCR in this study are indicated by blue lines. **b** Expression profiles of *MITF-A*, *MITF-H* and *MITF-M* during cochlear development were examined by reverse transcription-PCR. *MITF-M* expression was present at detectable levels in the *Mitf* ^*R/R*^ cochlea, but not in the *Mitf* ^*r/r*^ cochlea. **c** Immunoblotting analysis of *MITF* isoforms in the cochlea and skin. *MITF-M* expression was detectable in the *Mitf* ^*R/R*^ cochlea and skin, but not in samples from the *Mitf* ^*r/r*^ pigs. **d** Differential level of expression of the *Mitf* exons in *Mitf* ^*R/r*^ and *Mitf* ^*r/r*^ stria vascularis (SV). The M-exon showed a 11.5-fold decrease in the *Mitf* ^*r/r*^ SV. The fold change of each exon is estimated by comparing the normalized read count of each exon between *Mitf* ^*R/r*^ and *Mitf* ^*r/r*^ SV in the RNA-seq assay. Raw data for this is provided in Additional file 5; sheet 4, Data of Mitf exon fold change

Because an association assay was incapable of further identifying the causative mutation, we next investigated the differential expression of *Mitf* between the mutant and wild-type pigs. In humans, at least seven transcript variants (encoding seven isoforms) with the same number of promoters and first exons have been identified [15]; in pigs, the *Mitf* gene has not yet been fully characterized and only one transcript has been identified [26]. Thus, the techniques of reverse transcription-PCR and 5’-rapid amplification of cDNA ends (commonly known as 5’-RACE) were used to investigate the transcript variants in the porcine *Mitf* gene. The transcript variants of *Mitf-m*, *Mitf-a* and *Mitf-h* were identified in the cDNA from the porcine inner ear (Fig. 3a).

Moreover, we found that the *Mitf-m* transcript was normally expressed in *Mitf* ^*R/R*^ cochlea, but not in *Mitf* ^*r/r*^ cochlea at any developmental stage (Fig. 3b). No obvious differences were found in the expression levels of *Mitf-a* or *Mitf-h* between the *Mitf* ^*R/R*^ and *Mitf* ^*r/r*^ cochlea (Fig. 3b). RNA-seq assay was used to detect differences between the transcriptome profiles of the *Mitf* ^*R/r*^ and *Mitf* ^*r/r*^ SVs at embryonic day 85; at this time in the development, melanocytes remained in the *Mitf* ^*r/r*^ SVs (Additional file 1: Figure S3). A total of 28 genes showed two-fold differential expression, including some genes with known functions related to pigmentation and melanogenesis, but the *Mitf* gene was not among them (Additional file 4: Table S9). Because the algorithm used for calculating the gene RPKM values (reads per kilobase per million mapped reads, which serve to estimate gene expression) cannot separate the expression of *Mitf-m* from other equally expressed transcript variants, we obtained the normalized read count for each exon individually, and found that expression of the M-exon in wild-type SVs was approximately 11.5-fold higher than in mutant SVs (Fig. 3d). In addition, RNA-seq data indicated that expression of some marker genes of melanocytes or the SV intermediate cells remained in the *MITF* ^*r/r*^ SVs; these genes included S100, KIT, MUM1, and Kir1.2 (Additional file 4: Table S10). Most of the genes expressed in *MITF* ^*r/r*^ SVs, however, did not show significantly lower expression than the genes in the *MITF* ^*R/r*^ SVs. When we considered these results along with those from TEM analysis of prenatal SVs (Additional file 1: Figure S3), we determined that the intermediate cells remained in the *MITF* ^*r/r*^ SVs at the embryo stage and disappeared around birth. Thus, the expression differences between *MITF* ^*R/r*^ and *MITF* ^*r/r*^ SVs that we observed were indeed caused by *Mitf* mutation, rather than a lack of intermediate cells.

Subsequent immunoblotting assay showed an undetectable level of polypeptides of 55–70 kDa (the reported size range of the *MITF-M* isoform in melanoma [27]) in the *Mitf* ^*r/r*^ cochleae (Fig. 3c). Consistent with the previous data, the levels of *MITF-A* and *MITF-H* detected by immunoblot (Fig. 3c) were similar between the *Mitf* ^*R/R*^ and *Mitf* ^*r/r*^ cochleae. These results indicated that the expression of *MITF-M* was eliminated in the *Mitf* ^*r/r*^ pigs at both the transcript and protein levels, and this differential expression pattern itself indicated the existence of regulatory mutations in M isoform-specific regions. Consistently, eight of the 26 co-segregated variants were located in the M isoform-specific promoter (M-promoter; Additional file 4: Table S8).

### Transcriptional activity analysis of the *MITF* M-promoter

**Fig. 4:**
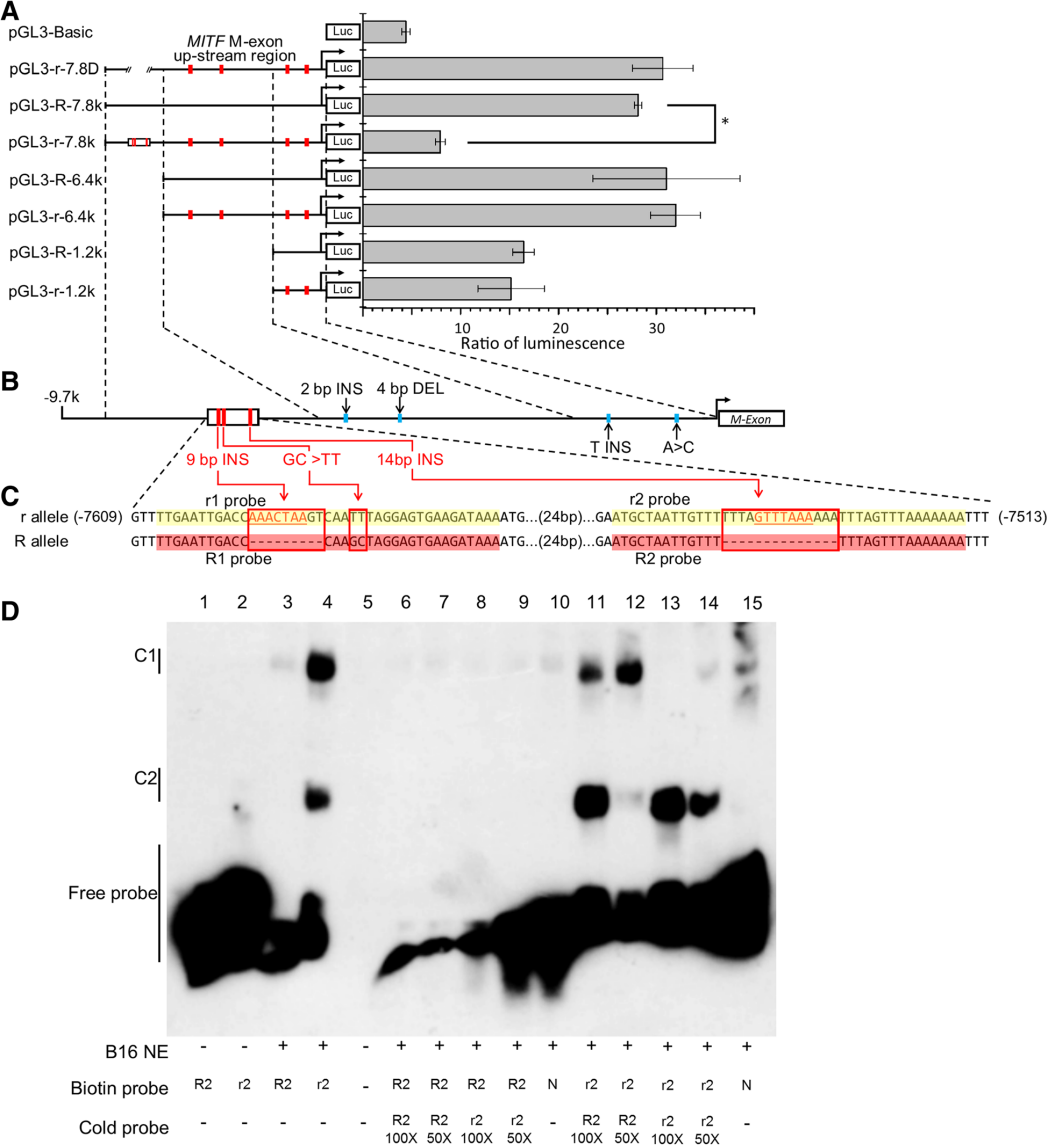
A new generated silencer in the M-promoter eliminated *Mitf-m* transcription. **a** Transcriptional activity analysis of the *Mitf-m* promoter from the R and r alleles. The reporter constructs are shown on the left, and the corresponding relative luciferase activity measured in transient transfection assays is shown on the right. The luciferase activity of pGL3-r-7.8 k was significantly lower than that of pGL3-R-7.8 k. There was no significant difference between the R and r alleles when constructs were shorter than 7.8 k. Error bars indicate the standard deviations. The results shown are for one experiment with four technical replicates. Raw data for this and three additional experiments with similar results are provided in Additional file 5; sheet 5, Data of reporter assay. **b** Schematics of the M-promoter. The sequence variations between the R and r alleles are labeled. INS, insertion; DEL, deletion. **c** Schematics of the M-promoter from –7513 bp to –7609 bp relative to the transcription start site of the M-exon. Sequence differences between R and r allele are indicated with a red box. The new sites showing consensus sequence for SOX protein binding are underlined in red. The oligonucleotide probes designed for electrophoretic mobility shift assay (EMSA) are highlighted. **d** EMSA shows the specific binding of the nuclear proteins to the r2 probe, and absence of binding to the R2 probe. In vitro incubation was performed using the indicated nuclear extracts, probe and unlabeled oligonucleotides (cold probes). C1, complex 1; C2, complex 2; R, R2 probe; r, r2 probe; N, random (negative control) probe

To test whether the variants in the M-promoter were capable of altering transcriptional activity, a transient transfection assay was performed using mouse B16 melanoma cells. The luciferase reporter constructs contained varying lengths (7.8, 6.4, 5.2, 3.7 or 1.2 kb) of truncated M-promoter from the R and r alleles, as shown in Fig. 4a. The construct pGL3-r-7.8 k, which contained a 7852-bp promoter region of the r allele, exhibited significantly lower luciferase activity than the R allele construct (pGL3-R-7.8 k; Fig. 4a). There was no significant difference in activity between the pGL3-r-7.8 k construct and the null construct (pGL3-Basic vector), the pGL3-R-6.4 k and pGL3-r-6.4 k constructs, or the pGL3-R-1.2 k and pGL3-r-1.2 k constructs (Fig. 4a). Together, these results suggested that the sequence variations involving the sequences between –7852 and –6416 bp, relative to the transcription start site of *Mitf-m*, were responsible for the elimination of *MITF-M* expression. Four co-segregated variants were located within that region, including two insertions (9 and 14 bp, respectively) and two continuous SNPs (Fig. 4b, c, red box). The mutations were densely clustered within a 96 bp region (Fig. 4a, b, black box). To further validate their effects on transcription, we knocked out the 96-bp fragment from the pGL3-r-7.8 k construct (named pGL3-r-7.8D) and detected restoration of the transcriptional activity (Fig. 4a). Using TFSEARCH [28], a transcription factor binding site searching tool, we predicted that the 9- and 14-bp insertions would create two putative binding sites for SOX family proteins (Fig. 4c, red underlined in red). As SOX proteins can act as suppressors of gene expression [29], we speculated that these insertions and the SNPs could be functional mutations.

To address whether these candidate mutations were able to alter the protein binding landscape in the involved sequence region, we used electrophoretic mobility shift assay (EMSA) to compare the binding capacity of the wild-type and mutant sequences. Two sets of oligonucleotides, R1 and r1 (Fig. 4c, red and yellow highlight, respectively), which differed only in the 9-bp insertion and the GC>TT replacement, and R2 and r2, which differed only in the 14-bp insertion (Fig. 4c, Additional file 4: Table S11), were incubated with nuclear extracts from mouse B16 melanoma cells. Only one differential complex (C1 in Fig. 4d) was formed, namely that with the r2 probe and lacking the R2 probe. The specificity of the complex was confirmed by competition EMSA, wherein a 50- and 100-fold molar excess of unlabeled r2 probe showed effective binding competition but a 50- and 2100-fold excess of unlabeled R2 probe did not. Another complex (C2 in Fig. 4d) formed was a non-specific complex, because no competition was observed with even 100-fold molar excess r2 cold probe. No difference in protein binding was observed between the R1 and r1 probes (Additional file 1: Figure S4). Collectively, these results show that only the 14-bp insertion can induce specific transcription factor binding events, while the 9-bp insertion and the two continuous SNPs do not. Thus, the 14-bp insertion is the only variation detected in our study that was considered as potentially responsible for the observed down-regulation of *Mitf-m* transcription activity.

SOX proteins (SOX2, SOX3 and SOX9) can regulate inner ear development [30–32]. Our RNA-seq data showed that *Sox2* and *Sox9*, but not *Sox3*, were expressed in porcine SVs (Additional file 4: Table S12), suggesting that SOX2 and/or SOX9 is capable of binding ectopically to a new negative CRE (i.e. a silencer), which may have been generated by the 14-bp short insertion in the *Mitf* regulatory region and which may have caused the abrogation of *Mitf-m* expression (Fig. 6). Thus, the phenotypes of hearing loss and depigmentation in the albino pigs appear to be caused, at least partially, by the 14-bp insertion that is located at –7532 bp relative to the transcription start site of *Mitf-m*. Additionally, we performed a further genotyping analysis of the causative mutant in 311 individual Rongchang pigs (Additional file 1: Figure S5). Both the 14- and 9-bp insertions were found to be carried by all albino pigs, and to co-segregate with the albino phenotype completely (Additional file 4: Table S13). No recombination event involving the two insertions was observed.

These results provide convincing evidence that even a small insertion in a region that lacks regulatory activity in the melanocyte lineage can create a transcription factor binding site (TFBS). Therefore, we next investigated whether this region was a non-regulatory sequence in other cell/species lineages. By exploiting the available data from the human ENCODE project, which had previously identified large numbers of regulatory elements, we were able to obtain the human ortholog of the mutant region identified in pigs and investigate whether this region overlapped with the CREs reported in the ENCODE data (Additional file 1: Figure S6A). The published RNA-seq data indicated that the wild-type M-exon was indeed transcribed in the human melanocyte lineage (Additional file 1: Figure S6C).

Using the UCSC browser to search the ENCODE data, we noted the following characteristics of these putative CREs. (1) The flanking regions of the causative mutant point has a relatively low level of conservation in mammals, suggesting a low probability of conserved functional CREs (Additional file 1: Figure S6B). (2) DNAse I hypersensitivity data indicate that chromatin accessibility around the mutant point is low in melanocytes and various cell lineages (Additional file 1: Figure S6D). (3) Chromatin immunoprecipitation and DNAse I footprinting data provide no evidence of protein binding sites near the mutant point (Additional file 1: Figure S6E). (4) The H3K4Me1 and H3K27Ac histone markers show no evidence of CREs in the flanking region of the mutant point (Additional file 1: Figure S6F). Because none of these data supported the existence of CREs near the mutant point, we concluded that the 14-bp insertion found in the albino pigs resulted in the de novo genesis of a silencer in the M-promoter (Fig. 6).

### *Mitf-m*-specific mutations result in hearing loss in a mouse model

**Fig. 5:**
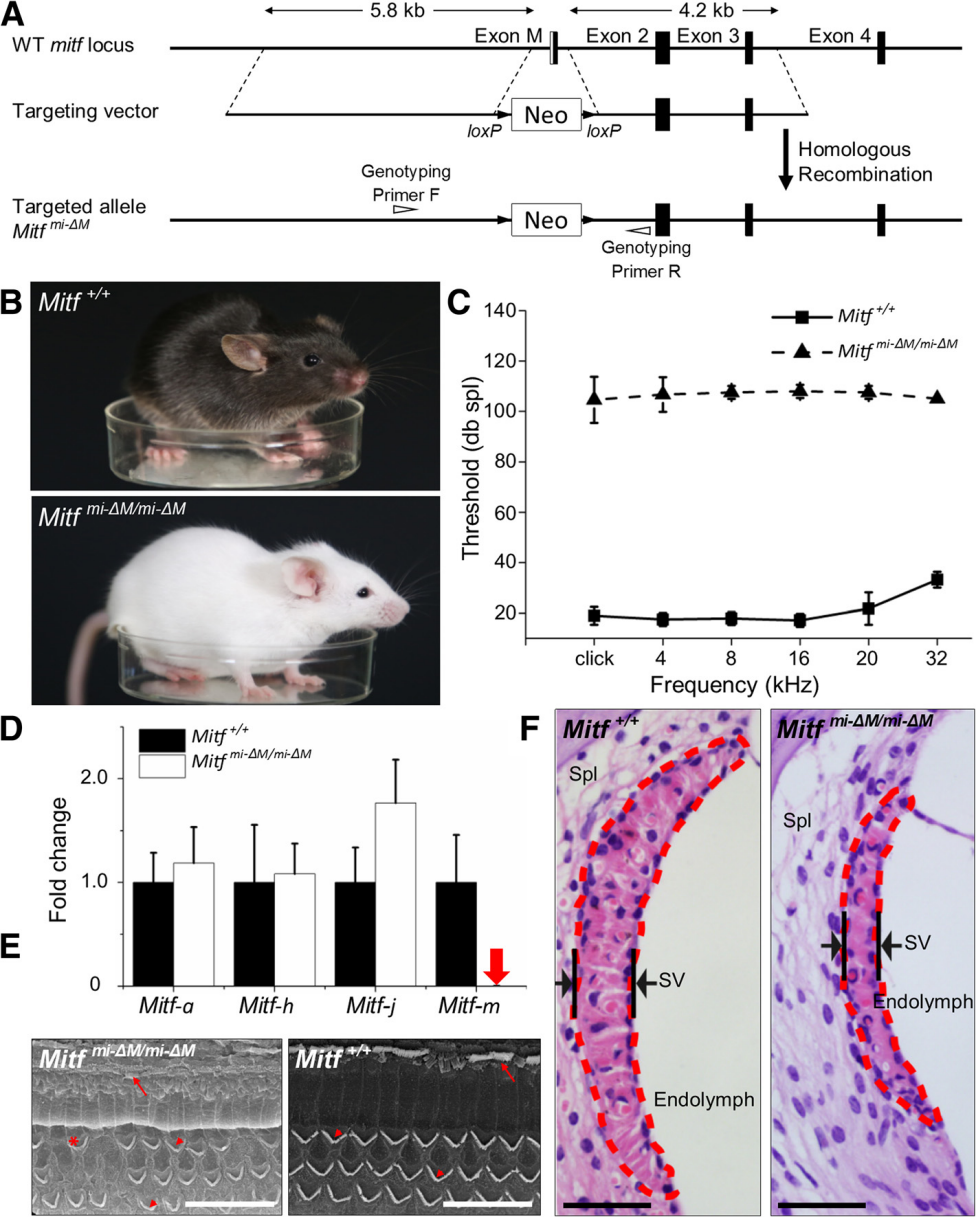
Phenotypes of the *Mitf-m* knock-out mice. **a** Schematic of the *Mitf-m* targeting technical process. The region of the *Mitf* gene containing exons M, 2, 3, and 4 are shown at the top. The targeting vector with a floxed-neomycin cassette in the M-promoter/M-exon region is shown in the middle. The resultant *Mitf* gene portion after targeting (*Mitf* ^*mi-ΔM*^ allele) is shown at the bottom. **b** *Mitf* ^*+/+*^ had a black coat color, and *Mitf* ^*mi-ΔM/mi-ΔM*^ had a white coat color and black eyes. **c** The auditory brainstem response thresholds were 20–30 dB SPL for the *Mitf* ^*+/+*^ mice, and 100–110 dB SPL for the *Mitf* ^*mi-ΔM/mi-ΔM*^ mice (from 4 to 32 kHz). The raw data is provided in Additional file 5: sheet 6 Data of ABR tests (mice). **d** *Mitf-m* was not expressed at detectable levels in the *Mitf* ^*mi-ΔM/mi-ΔM*^ cochlea (red arrow), but was expressed at detectable levels in the *Mitf* ^*+/+*^ cochlea. There was no difference observed between the expression levels of *Mitf-a* and *Mitf-h* in *Mitf* ^*mi-ΔM/mi-ΔM*^ and *Mitf* ^*+/+*^ mice. Error bars in **c** and **d** indicate the standard deviations. Raw data in Additional file 5: sheet 7 Data of mouse Mitf qPCR (Ct) and sheet 8 Data of mouse Mitf qPCR (FC). **e** In the *Mitf* ^*mi-ΔM/mi-ΔM*^ cochlea, most of the stereocilias of inner hair cells (arrows) and outer hair cells (arrowheads) were fused or missing (stars). **f** The stria vascularis of *Mitf* ^*mi-ΔM/mi-ΔM*^ cochlea are significantly thinner and shorter than that of *Mitf* ^*+/+*^ cochlea

To investigate whether a loss-of-function mutation in *Mitf-m* is sufficient to cause the deafness and depigmentation phenotypes, we constructed a mouse model with null *Mitf-m* alleles (*Mitf* ^*mi-ΔM/mi-ΔM*^ mouse; Fig. 5a). A quantitative PCR assay confirmed that the expression of *Mitf* transcriptional variants in the *Mitf* ^*mi-ΔM/mi-ΔM*^-targeted mouse was similar to those detected in the albino pigs (Fig. 5d). The *Mitf* ^*mi-ΔM/mi-ΔM*^ mice displayed profound hypopigmentation, with white hair and skin (Fig. 5b). ABR testing also revealed profound hearing loss in the *Mitf* ^*mi-ΔM/mi-ΔM*^ mice (n = 10; Fig. 5c). Finally, the *Mitf* ^*mi-ΔM/mi-ΔM*^ mice showed thinner SVs, compared to the wild-type mice, and fused or missing stereocilias of hair cells (Fig. 5e, f), similar to the cochlear morphology seen in the albino pigs. Together, these data demonstrate that an exclusive malfunction in the *m* transcript isoform is sufficient to cause the auditory-pigmentary phenotypes.

## Discussion

Genetic variations can create regulatory elements. Within the 10 million years of evolution involving modern organisms, hundreds of non-functional sequences have gained de novo regulatory functions [33]. Induced de novo *cis*-regulatory behaviors have been reported in the literature as having emerged via the following four genetic mechanisms. (1) Duplicated coding genes may lose their coding functions and gain de novo regulatory functions through a whole-genome duplication event; these de novo enhancers are consequently known as “recycle regions” [10]. (2) Point mutations can create new regulatory behaviors and are commonly observed, from flies to primates [6, 34], indicating their evolutionary benefit. (3) Deletions can also generate new enhancers by bringing together flanking sequences that create novel binding sites for activators [7]. (4) Transposons can serve as a source for acquiring new regulatory behaviors [8, 9, 35]. However, to our knowledge, short insertions acting as the source of de novo CREs have not been reported previously. Thus, our results provide evidence that short insertions can also create systemic functional silencers by introducing novel binding sites.

Previous studies have shown that transposons containing existing TFBS can facilitate de novo regulatory behavior and increase the transcriptional activity of target genes [9, 35]. In the current study, we found a de novo silencer in a distal melanocyte-specific regulatory region that had been naturally created by short insertions. Moreover, this de novo silencer acted as a negative CRE, blocking the expression of *Mitf-m*. This novel enhancer feature, introduced by an insertion event, comprised an in silico predicted SOX protein-binding consensus sequence. Our evidence indicated that a transcription factor (predicted to be SOX2 or SOX9) interacts with this novel binding site to repress the expression of *MITF-M*. Both the 9-bp and the 14-bp insertions were predicted to create new TFBS, and although only the 14-bp insertion interacted directly with nucleoproteins in vitro, we cannot rule out the possibility that the 9-bp insertion is the causative mutation, because the distance between the two insertion regions is as close as 50 bp and the HMG binding domain of SOX family proteins can bind to a sequence as large as 100 bp [36]. Thus, the 9-bp insertion region may act as a flanking sequence, affecting the robustness of the de novo element [37, 38].

Previous studies of the coding and regulatory regions of the *Mitf* gene have identified various functional mutations associated with congenital hearing loss in mammals [13, 20, 23, 24, 39]. Most of these mutations are located in the exons and introns that are conserved between species, and have been shown to cause equal genetic effects on multiple isoforms. Dysfunction in melanocytes in the SV is the major pathology of WS2. Apart from *MITF-M*, isoforms *MITF-A* and *MITF-H* are also expressed in melanocytes and melanoma cells [40–42]. The particular function(s) of each isoform in hearing loss remains to be fully elucidated. In a previous study of *Mitf*^*mi-bw*^ mice, insertion of a retrotransposing L1 element into intron 3 of the *MITF* gene abolished *MITF-M*, but also affected the expression of *MITF-A* and *MITF-H* [43]. Thus, the phenotypes on hearing loss and pigmentation cannot be exclusively attributable to the elimination of *MITF-M*, and there remains a possibility that *MITF-A* or *MITF-H* may play functional roles in the pathogenesis of WS2 and its manifested symptoms. Our study mapped a novel mutation located in the *MITF-M*-specific regulatory region that completely eliminated *MITF-M* expression, but which apparently had no effect on any other isoform (Fig. 3b, c). A search of the literature revealed that mutations in this region have also been shown to affect coat color in dogs [21] and horses [22]. Furthermore, we generated the first M-exon specific knock-out mouse model, we found phenotypes that were consistent with *Mitf*^*r/r*^ pigs, as well as similar *Mitf* expression profiles. Considering the collective results from the specific naturally-arising porcine mutation and the artificially-induced mouse mutation, it appears that *MITF-M* exerts a unique function in the inner ear and that dysfunction of the *MITF-M* isoform alone is sufficient to cause deafness.

*Mitf* is a well-described and frequent causative gene of WS2 [11, 12]. Most of the mutations found in *Mitf* are located in the coding region and the flanking splice donor/acceptor sites, accounting for the phenotype of hearing loss in approximately 15 % of WS2 cases; however, the mutation profile has not been determined in approximately 70 % of WS2 patients [13]. Our results suggest that the upstream region of the M-exon, which serves to regulate the expression of *Mitf-m*, is a novel potential mutation region in WS2 patients. Identifying and characterizing such regulatory regions will have clinical implications for identifying causative mutations for genetic diseases such as WS2. New techniques, such as targeted, capture-based sequencing and whole-genome re-sequencing, provide the possibility of mutation screening for large-scale regulatory regions. Finally, genetic hearing loss is almost exclusively studied in mouse models. The lack of a large animal model for genetic hearing loss has impaired the development of gene therapy for clinical application. Our *Mitf* ^*r/r*^ pigs should serve as a valuable model to be tested for such therapeutic intervention both by traditional gene therapy and by new CRISPR/Cas9-mediated genome editing [44].

## Conclusions

**Fig. 6:**
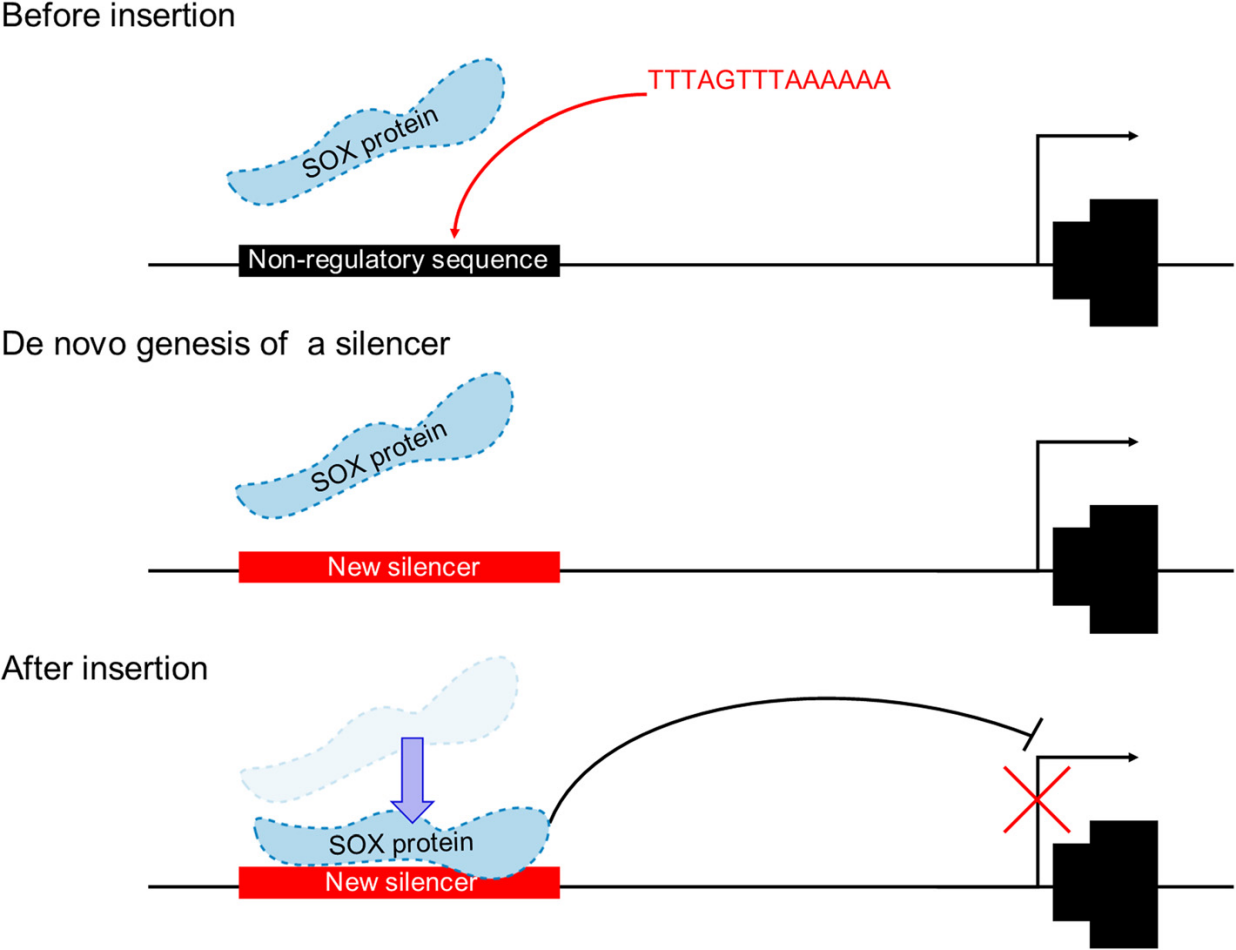
Schematics showing the genetic effect of the causative mutation. In *Mitf* ^*R/R*^ stria vascularis (SVs) (before duplication). SOX proteins cannot recognize and bind to the M-promoter and *MITF-M* was normally transcribed. In *Mitf* ^*r/r*^ SVs, a new consensus site for SOX protein binding, which resulted from the 14-bp duplication, created a de novo silencer in the M-promoter. SOX proteins ectopically binding to that silencer may repress the transcription of *Mitf-m* (after duplication)

In summary, we provide evidence, for the first time, that short insertions in non-coding regions, previously lacking *cis*-regulatory activity, are capable of creating systemic functional de novo CREs, resulting in dramatic phenotypic alterations in mammals (Fig. 6). We also identified the essential role of *MITF-M* in cochlear development and demonstrated that loss-of-function mutations of *MITF-M* are sufficient to cause deafness. Thus, it is important to include the regulatory regions in clinical gene screening for WS2.

## Methods

### Study population of Rongchang pigs

Chinese Rongchang pigs were chosen from the animal breeding facility of the Chongqing Academy of Animal Science. Pigs from 1 month to 1.5 years old (male and female) were used. All Rongchang pigs underwent auditory electrophysiology diagnoses to determine the hearing phenotype. Pigs with normal hearing were assigned to the control group and albino pigs with no ABR response were used as the case group. Use of the pigs and mice was approved by the Institutional Animal Care and Use Committee of China Agricultural University in Beijing.

### Auditory electrophysiology

ABR, EP and [K^+^] recording were used to evaluate hearing function of pigs and mice. Details of these tests have been described in our previous publications [45]. Briefly, ABRs were evoked with clicks and tone pips at 4, 8, 16, and 20 kHz. The ABR threshold was determined by visual inspection. EP and [K^+^] in the endolymph were recorded using double-barreled microelectrodes filled with 150 mM KCl and a potassium ion exchanger. The reference barrel was filled with 0.5 M NaCl for EP recording and the electrodes were calibrated in a 37 °C water bath chamber using a series of solutions of KCl and NaCl that had [K^+^] of 1, 5, 25, 50, 100, and 150 mM. At least four independent animals were tested to confirm the results.

### Cochlear morphology

The morphology of the stereocilia of cochlear hair cells was examined using SEM. Samples for SEM were prepared following procedures described in our previous publications [46]. Briefly, the cochleae were fixed with 2.5 % glutaraldehyde. After dehydration, the samples were critical-point dried, mounted on aluminum stubs, sputter-coated with gold particles, and examined using a Hitachi S-3700N SEM (Japan). The cochlear SV morphology was observed on cochlear sections using light microscopy and TEM. For TEM, the cochleae after overnight fixation were decalcified, embedded in Epon resin, and sectioned on a Reichert Ultracut E ultramicrotome (Boeckeler Instruments, Tucson, NM). Ultrathin sections were mounted on formvar-coated slot grids, stained with lead citrate and uranyl acetate, and examined using a Philips CM 120 TEM. At least three independent samples were performed to confirm the results.

### Genetic analysis and whole genome association study

Eleven pairs of putative heterozygous boars and sows were selected from the normal herd if they had albino offspring. In total, 74 piglets from 11 litters of heterozygous × heterozygous matings were phenotyped to investigate the Mendel segregation ratio. DNA from 28 pigs showing profound hearing loss and 73 pigs with normal hearing was isolated from tissue samples taken from pinnas. The DNA samples were genotyped using the Illumina PorcineSNP60 BeadChip. The PLINK software package was used for genome-wide mapping; all SNP markers with MAF > 0.05 and call rate > 75 % were used for a case-control association analysis. Genome-wide *P* values were ascertained through phenotype permutation testing (n = 100,000).

### Mutation screening

All exons, exon-intron boundaries and promoters (10-kb fragment upstream of TSS) were sequenced in 12 normal pigs and 12 albino pigs using the primers listed in Additional file 4: Table S11. Long-range PCR with LongAmpTaq DNA polymerase (NEB) was used to amplify these fragments. PCR fragments were gel-purified with an EZNA Gel Extraction kit (Omega Biotek) and then subjected to Sanger sequencing.

### Expression analysis

The mRNA expression levels of *MITF* transcript variants and β-actin were analyzed by a qPCR method using the primers in Additional file 4: Table S11. The cDNA samples (100 ng) and primers for the target genes were mixed with Power SYBR Green PCR Master Mix (Applied Biosystems) in 25-μL final volumes, and amplified using an ABI7900 instrument (Applied Biosystems). All samples were analyzed in triplicate. Protein expression levels of *MITF* isoforms and tubulin were examined by immunoblotting assays using lysed tissue from *MITF* ^*R/R*^ and *MITF* ^*r/r*^ cochleae or skin. MITF antibody C5 (#ab80651, Abcam) was used. The loading amounts were verified by determining levels of a housekeeping protein, β-tublin (Chemicon, Temecula, CA).

### Transcriptome analysis of SVs

The lateral walls of the cochlea containing the SVs were dissected from pigs, and preserved with the RNAlater reagent (Ambion, Austin, TX). Total RNA was extracted using the RNeasy Micro Kit (#74004, Qiagen, GRE). RNA concentration and integrity was measured using a Qubit 2.0 Flurometer (Life Technologies, CA, USA) and a Bioanalyzer 2100 system (Agilent Technologies, CA, USA), respectively. The IlluminaTruSeq RNA Sample Preparation Kit (Illumina, San Diego, USA) was used to generate sequencing libraries following the manufacturer’s recommendations. The clustering of samples was performed with the cBot Cluster Generation System according to the manufacturer’s instructions. Clustered libraries were sequenced on a Hiseq 2000 platform (Illumina) and 100-bp paired-end reads were generated. After quality control, clean reads with high quality were obtained for downstream analysis. Genome assembly Sscrofa10.2 was used as the reference genome for read mapping. Clean reads were aligned to the reference genome using TopHat (ver. 2.0.7) [33, 47]. RPKM values (reads per kilobase of exon model per million mapped reads) were calculated to measure the expression level of genes.

### Transcription activity analysis

The 7.8-kb fragments of the M-promoter of the R and r alleles were inserted into a luciferase reporter plasmid pGL3-basic (Promega, Madison, USA) to yield the pGL3-R-7.8 k and pGL3-r-7.8 k constructs, respectively. Similarly, truncated constructs including pGL3-R-6.4 k, pGL3-r-6.4 k, pGL3-R-1.2 k, and pGL3-r-1.2 k were generated. Murine B16 melanoma cells with high endogenous *MITF-M* expression were cultured for plasmid transfection. Luciferase reporter constructs (2.0 μg) and Renilla luciferase vector (0.5 μg) were co-transfected into B16 cells using Lipofectamine 2000 Transfection Reagent according to the manufacturer’s protocol (#11668-019, Invitrogen). At 48 h after transfection, luciferase activity was determined with a Dual-Luciferase Reporter Assay Kit (#E2920, Promega, Madison, USA). The Renilla luciferase activity was used to normalize the transfection efficiency. At least three independent experiments were performed.

### EMSA

Nuclear extracts from B16 cells were prepared using the Nuclear and Cytoplasmic Protein Extraction Kit (#P0027, Beyotime). Oligonucleotides representing the R and r allele fragments (Additional file 4: Table S11) were 3’ end-labeled with biotin and incubated with nuclear extract in the absence or presence of homologous unlabeled DNA (50-fold molar excess). The products were resolved by electrophoresis on an 8 % polyacrylamide gel with × 0.5 TBE at room temperature for 2 h at 150 V. At least three independent replicates were performed to confirm the results.

### *Mitf-m*-targeted mice

*Mitf* ^*mi-ΔM/mi-ΔM*^-targeted mice were generated using the “recombineering” technology. Briefly, a targeting construct with flanking regions (10,868 bp) of the M-promoter/M-exon was used for standard targeting of mouse embryonic stem cells. Positive cell clones were microinjected into eight-cell embryos to obtain chimeric mice. Chimeric mice that could transmit the modified *Mitf* m-exon allele to their progeny were crossed with wild-type mice to generate *Mitf* ^*mi-ΔM/+*^ mice. *Mitf* ^*mi-ΔM/mi-ΔM*^-targeted mice were obtained by *Mitf* ^*mi-ΔM/+*^ × *Mitf* ^*mi-ΔM/+*^ mating. Genotyping of the mice was performed by PCR using primers flanking the m-exon of *Mitf* (as shown in Additional file 4: Table S11). Use of the mice was approved by the Institutional Animal Care and Use Committee of the General Hospital of PLA in Beijing.

## Abbreviations

ABR, auditory brainstem response; CRE, cis-regulatory element; EMSA, electrophoretic mobility shift assays; EP, endolymphatic potential; MITF, microphthalmia-associated transcription factor; SEM, scanning electron microscopy; SNP, single-nucleotide polymorphism; SPL, sound pressure level; SSC 13, *Sus scrofa* chromosome 13; SV, stria vascularis; TEM, transmission electron microscopy; TFBS, transcription factor binding site; WGD, whole-genome duplication; WS2, Waardenburg syndrome type 2

## Additional files

Additional file 1: Figure S1. Eye morphology defects of albino pigs. **Figure S2.** Three family pedigrees of mapping population. **Figure S3.** Images showing presence of intermediate cells in the stria vascularis of albino pigs at the embryo stage. **Figure S4**. Results of EMSA using probe R1 and r1. **Figure S5.** Genotyping of Rongchang pigs for causative mutation. **Figure S6.** The human orthologous of the causative mutant region found in *MITF* ^*r/r*^ pigs are formerly lack of regulatory activity. (DOCX 6667 kb) Additional file 2: Table S1. The goodness of fit test for Mendelian ratios of the hearing loss. **Table S2.** Re-annotation of porcine *MITF* gene in Genome of Tibet pig. **Table S3.** Co-segregated mutations detected in mutation screening. **Table S4.** Summary and mapping statistics of the pig genome re-sequencing data. (DOCX 58 kb) Additional file 3: Table S5. SNPs detected by re-sequencing in the associated region of Rongchang pigs. **Table S6.** SNPs co-segregated with hearing loss phenotype in three *MITF* ^*r/r*^ pigs and three *MITF* ^*R/R*^ Rongchang pigs. **Table S7.** SNPs uniquely detected in Rongchang pigs, and homozygous in *MITF* ^*r/r*^ *. (XLSX 465 kb)*
Additional file 4: Table S8. Co-segregated variants detected in re-sequencing and mutation screening. **Table S9.** Differential expressed genes between *MITF* ^*R/r*^ and *MITF* ^*r/r*^ stria vascularis (SVs). **Table S10.** Expression levels of melanocyte marker genes in porcine SVs. **Table S11.** Primer pairs used for screening the *MITF* gene, for qPCR and for mice genotyping. **Table S12.** Expression levels of SOX family members in porcine SVs. **Table S13.** Distribution of hearing loss phenotype and genotype in a large Rongchang pig population. (DOCX 55 kb) Additional file 5:
**Supporting data.** 1 Data of ABR tests (pigs). 2 Data of EP. 3 Data of K+ concentration. 4 Data of Mitf exon fold change. 5 Data of reporter assay. 6 Data of ABR tests (mice). 7 Data of mouse Mitf qPCR (Ct). 8 Data of mouse Mitf qPCR (FC). (XLSX 27 kb)
