# Supplementary material for: A de novo silencer causes elimination of MITF-M expression and profound hearing loss in pigs
Source: BMC Biol. 2016 Jun 27;14:52. doi: 10.1186/s12915-016-0273-2 (PMC4922063; doi:10.1186/s12915-016-0273-2)
Supplement: Additional file 1: Figure S1. — Eye morphology defects of albino pigs. Figure S2. Three family pedigrees of mapping population. Figure S3. Images showing presence of intermediate cells in the stria vascularis of albino pigs at the embryo stage. Figure S4. Results of EMSA using probe R1 and r1. Figure S5. Genotyping of Rongchang pigs for causative mutation. Figure S6. The human orthologous of the causative mutant region found in MITF r/r pigs are formerly lack of regulatory activity. (DOCX 6667 kb) [file 12915_2016_273_MOESM1_ESM.docx]

**Supplementary Figure 1**


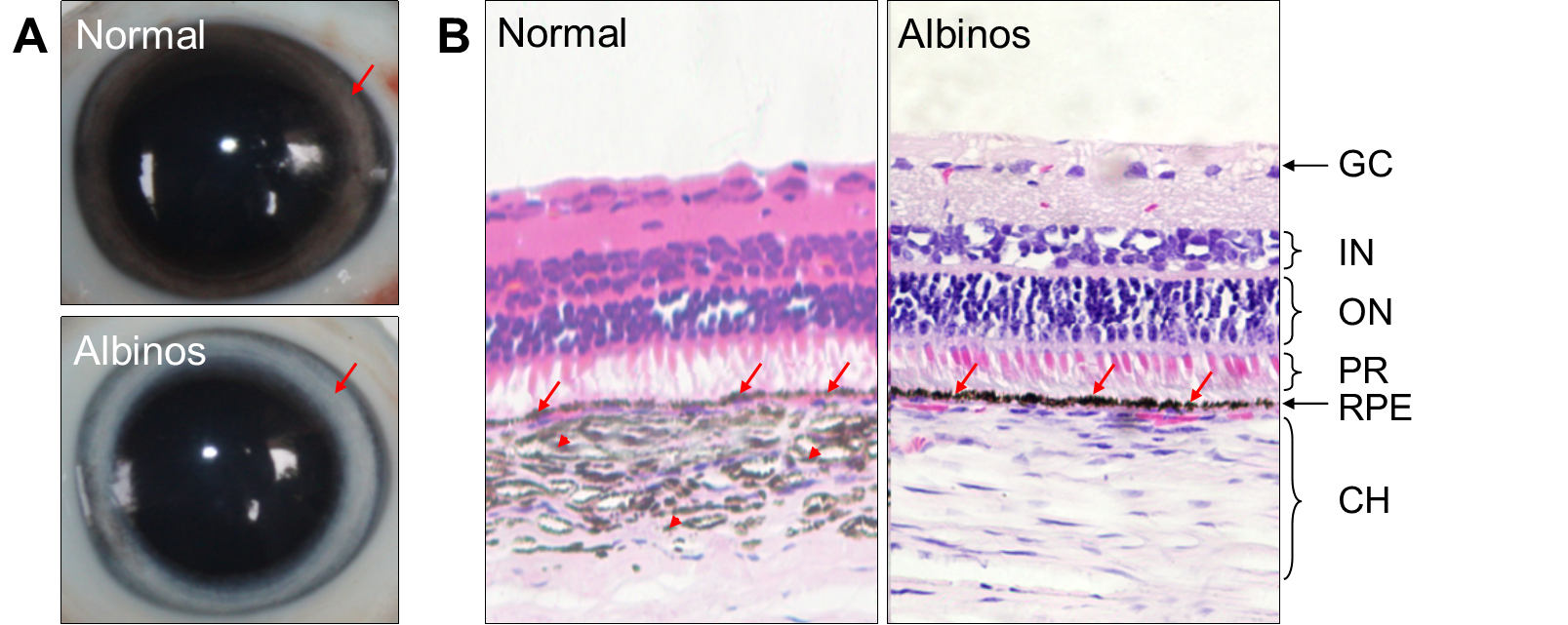


**Supplementary Fig. 1 |** Eye morphology defects of albino pigs. (A) Gross images of a normal eye and an albino eye. Iris, arrows. (B) Paraffin sections of a normal retinae and an albino retina. Ganglion cells, GC; inner nuclear layer, IN; outer nuclear layer, ON; photoreceptors, PR; retinal Pigment Epithelium, RPE; choroid, CH; melanin in choroid, arrowheads; melanin in RPE, arrow.

**Supplementary Figure 2**


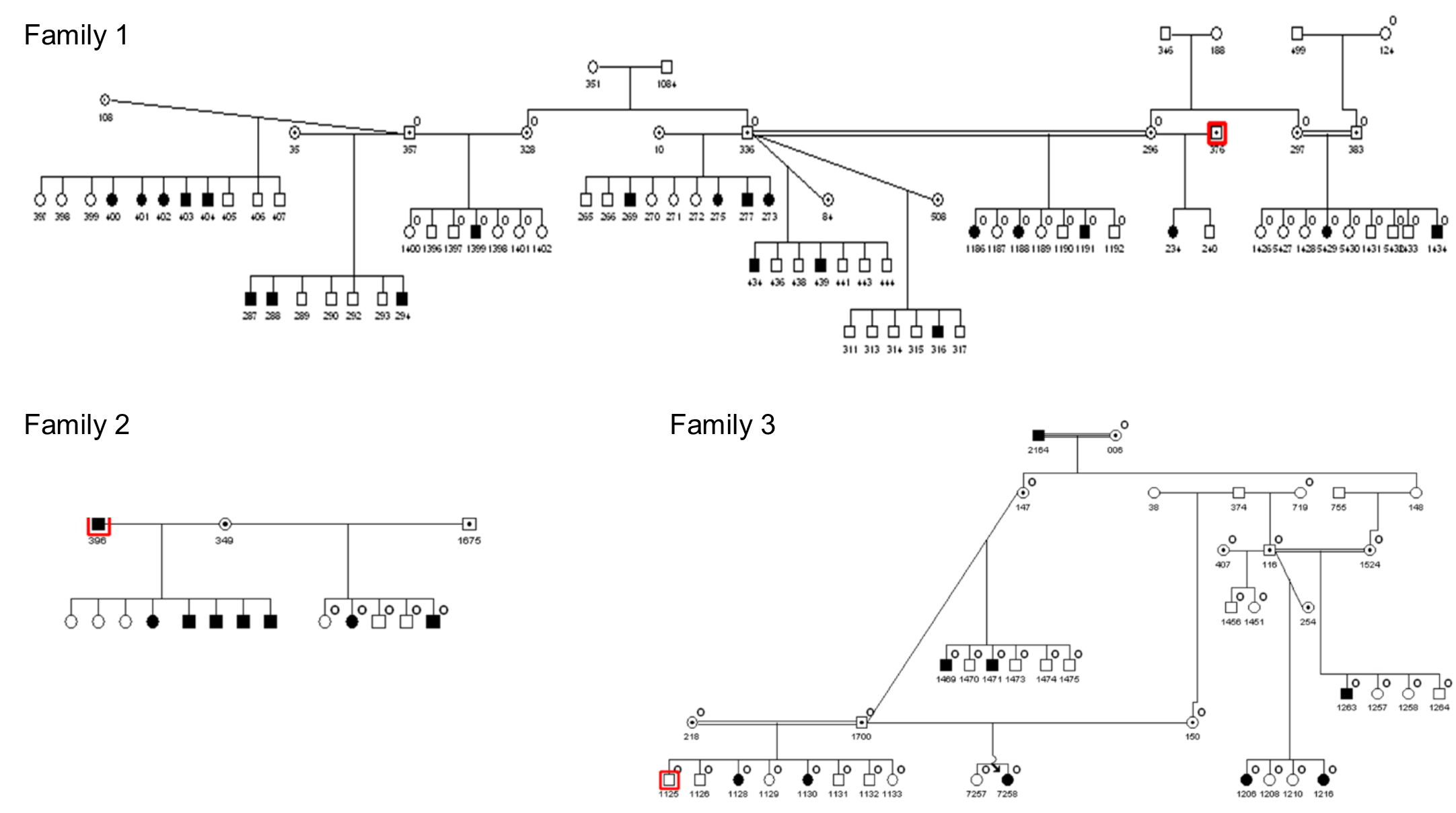


**Supplementary Fig. 2 |** Three family pedigrees of mapping population. The filled symbols represent deaf pigs and the open symbols represent pigs with normal hearing. DNA samples of the pigs marked on the up-right corner with circles have been collected for the whole genome typing.

**Supplementary Figure 3**


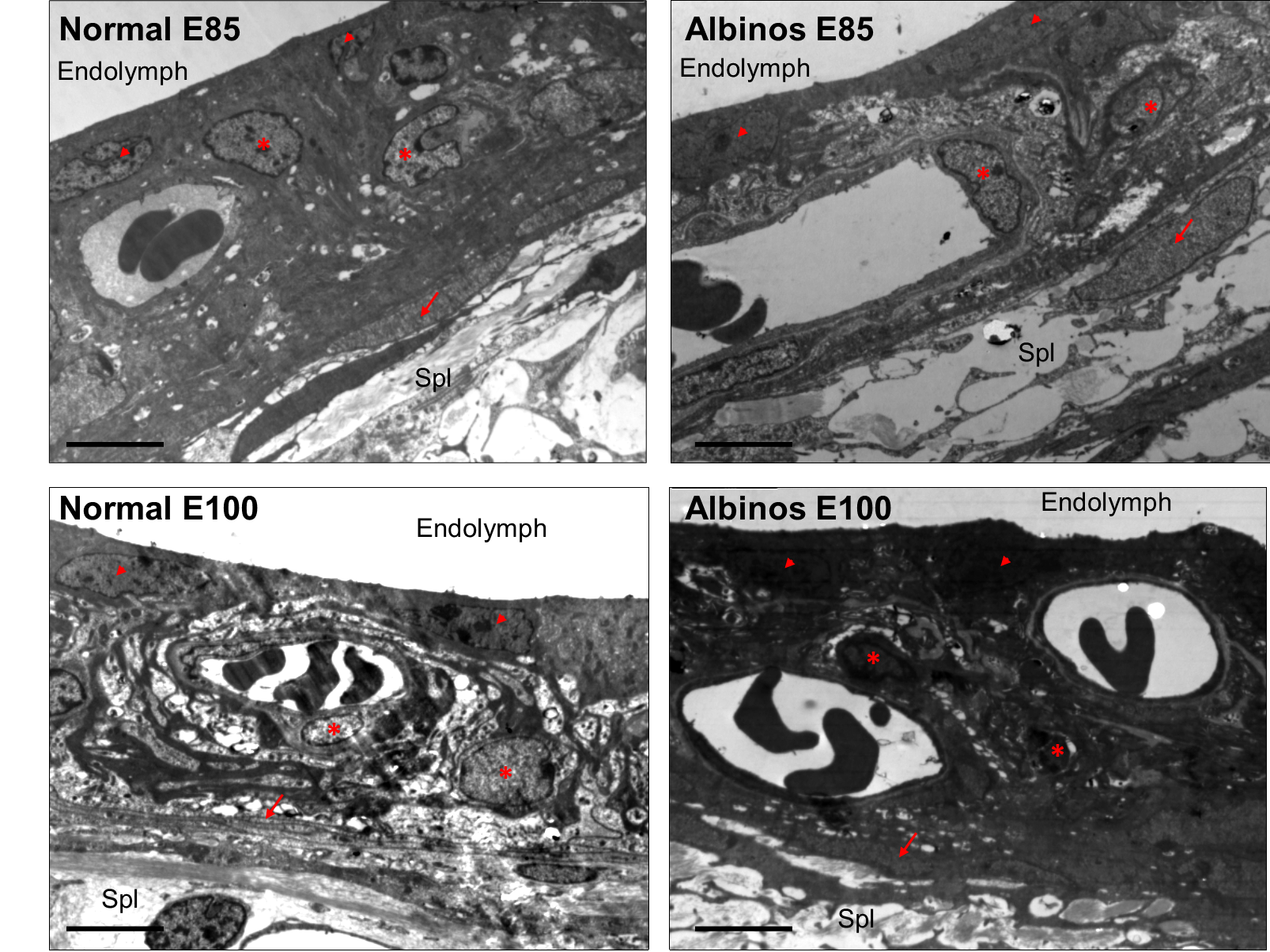


**Supplementary Fig. 3** | Images showing presence of intermediate cells in the SV of albino pigs at the embryo stage. marginal cell, arrowheads; intermediate cell, stars; basal cell, arrows; spiral ligament, Spl. Scale bars = 5 μm.

**Supplementary Figure 4**


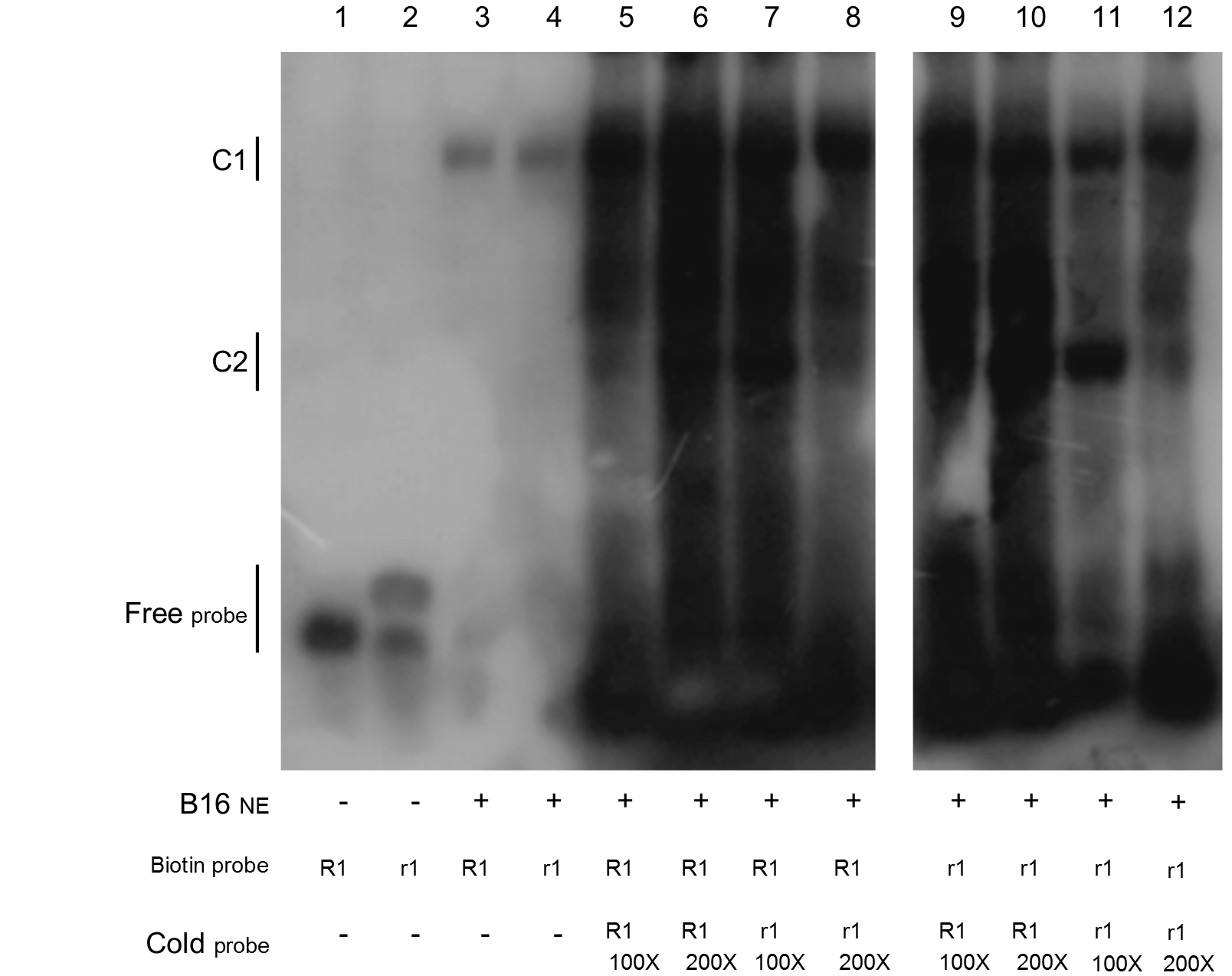


**Supplementary Fig. 4 |** Results of EMSA using probe R1 and r1. The protein binding behavior showed no difference between R1 and r1 probe when they were incubated with B16 nuclear extracts (NE). Cold probe, unlabeled oligonucleotides; C1, complex 1; C2, complex 2; R1, R1 probe; r1, r1 probe.

**Supplementary Figure 5**


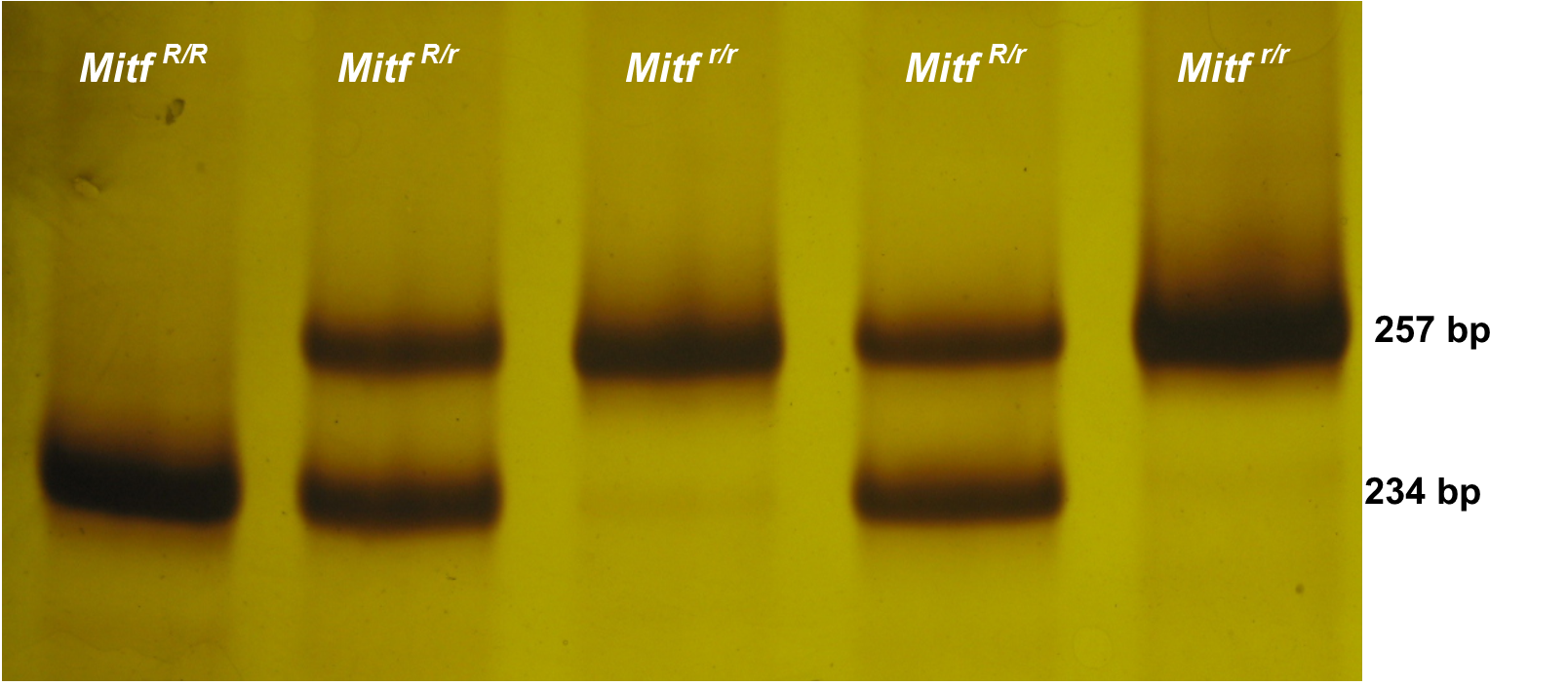


**Supplementary Fig. 5 |** Genotyping of Rongchang pigs for causative mutation. PCR based genotyping amplified a 234 bp fragment (lower band) located in 7.5 kb upstream of wild type M exon. The 9 bp and 14 bp insertions in mutant allele created a 257 bp amplicon (upper band).

**Supplementary Figure 6**


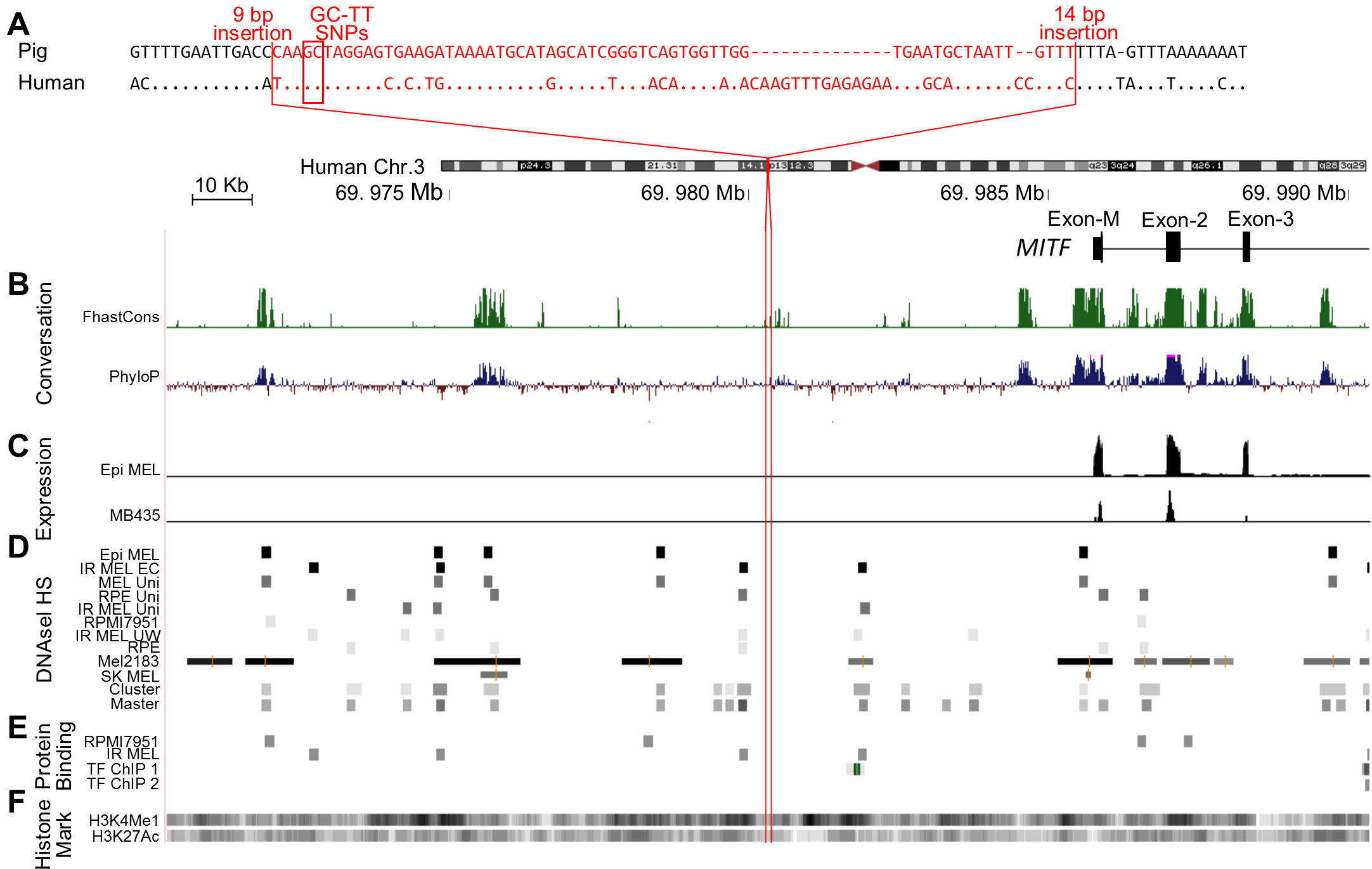


**Supplementary Fig. 6 |** The human orthologous of the causative mutant region found in *MITF^r/r^* pigs are formerly lack of regulatory activity. (a) Sequence alignment of causative mutant region between pig and human. The inserted point (red vertical line) and the replaced base pairs (red box) are denoted. (b) Measurements of evolutionary conservation of 46 mammal species. PhastCons and phyloP represent two algorithm of conservation scores across this region. (c) The transcription levels across this region in melanocytes and melanoma cells determined by ENCODE RNA-seq assay indicates the *Mitf-M* expression. Epi MEL: Plus signal of epidermal melanocyte (ENCODE accession number: ENCFF000IGD). MB435: Raw signal of MDA-MB-435 cell line which appears to have been contaminated with the M14 melanoma cell line (GEO accession number: GSM325488). (d) No active cis-regulatory sequences was detected in the causative mutant region by DNaseI hypersensitivity. Epi MEL: DNaseI hypersensitive peaks of epidermal melanocytes (ENCODE accession number: ENCFF000ADT). IR MEL EC: DNaseI hypersensitive peaks of iris pigment epithelial cells (ENCODE accession number: ENCFF001BFI). MEL Uni: Uniform set of DNaseI hypersensitive sites (DHSs) in epidermal melanocytes (UCSC accession number: wgEncodeEH000602). RPE Uni: Uniform set of DNaseI hypersensitive sites (DHSs) in retinal pigment epithelial cells (UCSC accession number: wgEncodeEH000517). IR MEL Uni: Uniform set of DNaseI hypersensitive sites (DHSs) in iris pigment epithelial cells (UCSC accession number: wgEncodeEH001184). RPMI7951: DHSs of RPMI7951 melanoma cells from University of Washington (UCSC accession number: wgEncodeEH003007). IR MEL UW: DHSs of iris pigment epithelial cells from University of Washington (UCSC accession number: wgEncodeEH001184). RPE: DHSs of retinal pigment epithelial cells from University of Washington (UCSC accession number: wgEncodeEH000517). Mel2183: DHSs of Mel2183 melanoma cells from Duke University (UCSC accession number: wgEncodeEH002557). SK MEL: DHSs of epidermal melanocytes from Duke University (UCSC accession number: wgEncodeEH000602). Cluster: DNaseI hypersensitivity clusters in 125 cell types (UCSC track label: DNase Clusters). Master: A summary of DHSs in 125 separate cell type (UCSC track label: Master DNaseI HS). (e) There is no evidence of protein binding was detected by DNaseI genomic foot printing (DGF) and ChIP-seq in the causative mutant region. RPMI7951: DGF peaks in RPMI7951 melanoma cells (UCSC accession number: wgEncodeEH003150). IR MEL: DGF peaks in iris pigment epithelial cells (UCSC accession number: wgEncodeEH002446). Txn Factor ChIP: Regions of transcription factor binding in 91 cell types derived from a large collection of ChIP-seq experiments performed by 161 factors of ENCODE Factorbook repository (UCSC track label: Txn Factor ChIP). Txn Fac ChIP V2: Regions of transcription factor binding in over 110 cell types derived from a large collection of ChIP-seq experiments performed by over 200 factors (UCSC track label: Txn Fac ChIP V2). (f) Histone marks of regulatory elements measured in this region by ChIP-seq. H3K4Me1: H3K4Me1 Mark (often found near regulatory elements) on 7 cell lines (UCSC track label: Layered H3K4Me1). H3K27Ac: H3K27Ac Mark (often found near active regulatory elements) on 7 cells lines (UCSC track label: Layered H3K27Ac).
