# Supplementary material for: A de novo silencer causes elimination of MITF-M expression and profound hearing loss in pigs
Source: BMC Biol. 2016 Jun 27;14:52. doi: 10.1186/s12915-016-0273-2 (PMC4922063; doi:10.1186/s12915-016-0273-2)
Supplement: Additional file 2: Table S1. — The goodness of fit test for Mendelian ratios of the hearing loss. Table S2. Re-annotation of porcine MITF gene in Genome of Tibet pig. Table S3. Co-segregated mutations detected in mutation screening. Table S4. Summary and mapping statistics of the pig genome re-sequencing data. (DOCX 58 kb) [file 12915_2016_273_MOESM2_ESM.docx]

**Supplementary Table 1** | The goodness of fit test for Mendelian ratios of the hearing loss phenotype

|  | Observed | Expected | O-E | X^2 a^ |
| --- | --- | --- | --- | --- |
| Controls | 49 | 55.5 | -6.5 | 2.59 |
| Cases | 25 | 18.5 | 6.5 |  |
| Total | 74 | 74 |  |  |

^a^ Exceeds critical value for chi-square tests, 3.84, df = 1. **Supplementary Table 2** | Re-annotation of porcine *MITF* gene in Genome of Tibet pig

| Exon No. | Sus scrofa | | | | | Homo sapiens | | | | |
| --- | --- | --- | --- | --- | --- | --- | --- | --- | --- | --- |
|  | Length | Ori | Scaffold | Start | End | Length | Ori | Chr. | Start | End |
| Exon A | 268 | + | 24421 | 11223 | 11490 | 267 | + | 3 | 69788586 | 69788852 |
| Exon O | 138 | + | 24421 | 34380 | 34481 | 137 | + | 3 | 69812707 | 69812843 |
| Exon C | 134 | + | 24421 | 34634 | 34767 | 132 | + | 3 | 69812962 | 69813093 |
| Exon H | 125 | + | 24421 | 145785 | 145908 | 123 | + | 3 | 69915375 | 69915497 |
| Exon B | 248 | + | 24421 | 159749 | 159996 | 250 | + | 3 | 69928285 | 69928534 |
| Exon M | 153 | + | 24421 | 218123 | 218275 | 156 | + | 3 | 69985751 | 69985906 |
| Exon 2 | 231 | + | 24421 | 219321 | 219551 | 228 | + | 3 | 69986973 | 69987200 |
| Exon 3X | 708 | + | 24421 | 219861 | 220568 | 714 | + | 3 | 69987503 | 69988216 |
| Exon 3 | 86 | + | 24421 | 220599 | 220684 | 84 | + | 3 | 69988249 | 69988332 |
| Exon 4 | 99 | + | 24421 | 223120 | 223218 | 96 | + | 3 | 69990387 | 69990482 |
| Exon 5 | 112 | + | 24421 | 231071 | 231182 | 118 | + | 3 | 69998202 | 69998319 |
| Exon 6 | 58 | + | 24421 | 234193 | 234250 | 57 | + | 3 | 70000981 | 70001037 |
| Exon 7 | 76 | + | 24421 | 241157 | 241232 | 76 | + | 3 | 70005606 | 70005681 |
| Exon 8 | 149 | + | 24421 | 244715 | 244863 | 148 | + | 3 | 70008424 | 70008571 |
| Exon 9 | 3586 | + | 24421 | 250987 | 254571 | 3491 | + | 3 | 70013998 | 70017488 |

**Supplementary Table 3** | Co-segregated variants detected in mutation screening.

| Position | Variants type | Ref. allele | Mutant allele | Location |
| --- | --- | --- | --- | --- |
| **56331126** | **SNP** | **G** | **A** | **Promoter O** |
| **56331350** | **SNP** | **A** | **C** | **Promoter O** |
| **56331444** | **SNP** | **A** | **T** | **Promoter O** |
| 56461308 | SNP | G | A | Intron 5 |
| **56469663** | **SNP** | **T** | **A** | **Intron 4** |
| 56469862 | SNP | G | C | Intron 4 |
| 56472689 | SNP | C | A | Exon 3 |
| 56474675 | SNP | G | A | Intron 1 |
| **56474762** | **SNP** | **A** | **T** | **Intron 1** |
| **56475668** | **SNP** | **T** | **A** | **Promoter M** |
| **56475832** | **SNP** | **A** | **C** | **Promoter M** |
| 56475912 | Insertion | A | AT | Promoter M |
| 56477462 | SNP | A | C | Promoter M |
| 56477466-56477470 | Deletion | GGGTA | G | Promoter M |
| **56477645** | **SNP** | **G** | **A** | **Promoter M** |
| **56477985** | **SNP** | **G** | **A** | **Promoter M** |
| 56481335 | Insertion | T | TAC | Promoter M |
| 56482632 | Insertion | T | TTTTAGTTTAAAAAA | Promoter M |
| 56482690 | SNP | C | T | Promoter M |
| 56482691 | SNP | G | T | Promoter M |
| 56482695 | Insertion | C | CAAACTAAGT | Promoter M |

**Supplementary Table 4** | Summary and mapping statistics of the pig genome re-sequencing data.

| Population | Pig name | Land of origin | Individual | High-quality base (Gb) | Depth (×) | Accession number |
| --- | --- | --- | --- | --- | --- | --- |
| Domestic pig (Chinese) | Rongchang | Rongchang city, Chongqing province, China | Rongchang1  (*MITF^R/R^* or *MITF^R/r^*) | 24.9 | 6.78 | SRX397138 |
|  |  |  | Rongchang2  (*MITF^R/R^* or *MITF^R/r^*) | 27.22 | 7.35 | SRX397141 |
|  |  |  | Rongchang3  (*MITF^R/R^* or *MITF^R/r^*) | 21.33 | 5.71 | SRX397139 |
|  |  |  | Rongchang4  (*MITF ^r/r^*) | 19.07 | 5.22 | SRX397137 |
|  |  |  | Rongchang5  (*MITF^r/r^*) | 26.15 | 6.86 | SRX397140 |
|  |  |  | Rongchang6  (*MITF^r/r^*) | 25.22 | 6.77 | SRX397142 |
|  | Penzhou | Luzhou city, Sichuan province, China | PZ1 | 11.83 | 3.6 | SRS387288 |
|  |  |  | PZ2 | 11.83 | 3.59 | SRS387286 |
|  |  |  | PZ3 | 14.07 | 4.15 | SRS387290 |
|  | Wujin | Liangshan Yi autonomous prefecture, Sichuan province, China | WJ1 | 15.88 | 4.55 | SRS387291 |
|  |  |  | WJ2 | 14.22 | 4.16 | SRS387294 |
|  |  |  | WJ3 | 12.01 | 3.58 | SRS387296 |
|  | Ya'nan | Chengdu city, Sichuan province, China | YN1 | 12.11 | 3.56 | SRS387298 |
|  |  |  | YN2 | 11.09 | 3.35 | SRS387299 |
|  |  |  | YN3 | 13.08 | 4 | SRS387302 |
|  | Neijiang | Neijiang city, Sichuan province, China | NJ1 | 15.73 | 4.13 | SRS387304 |
|  |  |  | NJ2 | 17.27 | 4.89 | SRS387306 |
|  |  |  | NJ3 | 11.42 | 3.46 | SRS387311 |
|  | Jinhua | Jinhua city, Zhejiang province, China | JH1 | 11.6 | 3.57 | SRS387315 |
|  |  |  | JH2 | 12.39 | 3.75 | SRS387317 |
|  |  |  | JH3 | 10.6 | 3.17 | SRS387319 |
|  | Meishan | Jiangsu province, China | Meishan1 | 18.03 | 6.35 | ERS177331 |
|  |  |  | Meishan2 | 17.92 | 6.23 | ERS177332 |
|  |  |  | Meishan3 | 17.17 | 5.64 | ERS177333 |
|  |  |  | Meishan4 | 19.76 | 7.22 | ERS177334 |
|  | Jiangquhai | Jiangsu province, China | Jiangquhai | 20.5 | 7.49 | ERS177311 |
|  | Xiang | Guangxi province, China | Xiang1 | 17.66 | 5.95 | ERS177355 |
|  |  |  | Xiang2 | 17.37 | 5.8 | ERS177356 |
| Domestic pig (European) | Duroc | Denmark, North American | Duroc1 | 21.01 | 5.53 | ERS177302 |
|  |  |  | Duroc2 | 22.69 | 6.46 | ERS177303 |
|  |  |  | Duroc3 | 11.74 | 4.24 | ERS177304 |
|  |  |  | Duroc4 | 14.76 | 5.37 | ERS177305 |
|  | Hampshire | England, North American | Hampshire1 | 22.51 | 6.28 | ERS177306 |
|  |  |  | Hampshire2 | 19.72 | 5.68 | ERS177307 |
|  | Landrace | Denmark | Landrace1 | 18.34 | 6.69 | ERS177312 |
|  |  |  | Landrace2 | 27.01 | 7.43 | ERS177313 |
|  |  |  | Landrace3 | 17.56 | 4.95 | ERS177314 |
|  |  |  | Landrace4 | 14.48 | 5.25 | ERS177315 |
|  |  |  | Landrace5 | 14.87 | 5.45 | ERS177316 |
|  | Large White | England | LW1 | 10.89 | 4.02 | ERS177317 |
|  |  |  | LW2 | 19.98 | 7.02 | ERS177318 |
|  |  |  | LW3 | 19.98 | 7.04 | ERS177319 |
|  |  |  | LW4 | 19.96 | 7.14 | ERS177320 |
|  |  |  | LW5 | 18.47 | 6.57 | ERS177321 |
|  |  |  | LW6 | 22.72 | 6.1 | ERS177322 |
|  |  |  | LW7 | 18.57 | 6.68 | ERS177323 |
|  |  |  | LW8 | 18.99 | 4.32 | ERS177324 |
|  |  |  | LW9 | 19.44 | 7.03 | ERS177325 |
|  |  |  | LW10 | 16.65 | 5.62 | ERS177326 |
|  |  |  | LW11 | 17.38 | 5.73 | ERS177327 |
|  |  |  | LW12 | 18.52 | 6.25 | ERS177328 |
|  |  |  | LW13 | 13.59 | 4.58 | ERS177329 |
|  |  |  | LW14 | 17.02 | 5.76 | ERS177330 |
|  | Pietrain | Belgium | Pietrain1 | 20.68 | 4.59 | ERS177336 |
|  |  |  | Pietrain2 | 20.91 | 7.61 | ERS177337 |
|  |  |  | Pietrain3 | 16.45 | 5.78 | ERS177338 |
|  |  |  | Pietrain4 | 10.88 | 3.98 | ERS177339 |
|  |  |  | Pietrain5 | 21.44 | 4.55 | ERS177340 |
| Wild boar (European) | France | France | France | 17.64 | 6.8 | ERS177349 |
|  | Switzerland | Switzerland | Switzerland | 28.39 | 5.84 | ERS177350 |
|  | Veluwe | Veluwe, the Netherlands | Netherlands 1 | 18.18 | 6.66 | ERS177345 |
|  |  |  | Netherlands 2 | 22.56 | 6.81 | ERS177346 |
|  | Meinweg | Meinweg, the Netherlands | NM1 | 10.56 | 3.89 | ERS177347 |
|  |  |  | NM2 | 15.7 | 5.67 | ERS177348 |
| Wild boar (Asian) | Japan | Japan | Japan | 21.55 | 7.84 | ERS177344 |
|  | North China | North China | NChina 1 | 9.31 | 3.4 | ERS177353 |
|  |  |  | NChina 2 | 19.29 | 7.01 | ERS177354 |
|  | South China | South China | SChina 1 | 9.83 | 3.62 | ERS177351 |
|  |  |  | SChina 2 | 19.83 | 7.2 | ERS177352 |
|  | Southwest China | Southwest China | WB1 | 12 | 3.41 | SRS387320 |
|  |  |  | WB2 | 16.3 | 4.71 | SRS387323 |
|  |  |  | WB3 | 16.28 | 4.65 | SRS387324 |
|  | Sumatran | Sumatra, Indonesia | Sumatra 1 | 21.56 | 7.72 | ERS177308 |
|  |  |  | Sumatra 2 | 20.98 | 7.68 | ERS177310 |
| Tibetan pig | Ganzi | Ganzi Tibetan autonomous prefecture, Sichuan province, China | T1-1 | 12 | 3.59 | SRS387185 |
|  |  |  | T1-2 | 12.15 | 3.6 | SRS387186 |
|  |  |  | T1-3 | 10.64 | 3.16 | SRS387190 |
|  |  |  | T1-4 | 14.29 | 4.26 | SRS387202 |
|  |  |  | T1-5 | 14.23 | 4.22 | SRS387204 |
|  | Diqing | Diqing Tibetan autonomous prefecture, Yunnan province, China | T2-1 | 16.05 | 4.71 | SRS387211 |
|  |  |  | T2-2 | 12.19 | 3.62 | SRS387213 |
|  |  |  | T2-3 | 11.78 | 3.51 | SRS387215 |
|  |  |  | T2-4 | 17.61 | 5.32 | SRS387217 |
|  |  |  | T2-5 | 11.64 | 3.54 | SRS387234 |
|  | Nyingchi | Nyingchi prefecture, Tibetan autonomous region, China | T3-1 | 9.78 | 2.63 | SRS387236 |
|  |  |  | T3-2 | 19.04 | 5.66 | SRS387238 |
|  |  |  | T3-3 | 13.4 | 3.95 | SRS387240 |
|  |  |  | T3-4 | 12.15 | 3.59 | SRS387241 |
|  |  |  | T3-5 | 17.87 | 5.33 | SRS387244 |
|  | Shigatse | Shigatse prefecture, Tibetan autonomous region, China | T4-1 | 14.7 | 4.37 | SRS387246 |
|  |  |  | T4-2 | 11.49 | 3.43 | SRS387248 |
|  |  |  | T4-3 | 15.05 | 4.42 | SRS387249 |
|  |  |  | T4-4 | 12.41 | 3.74 | SRS387251 |
|  |  |  | T4-5 | 14.87 | 4.43 | SRS387264 |
|  | Gannan | Gannan Tibetan autonomous prefecture, Gansu province, China | T5-1 | 15.56 | 4.65 | SRS387265 |
|  |  |  | T5-2 | 12.04 | 3.59 | SRS387268 |
|  |  |  | T5-3 | 12.94 | 3.81 | SRS387270 |
|  |  |  | T5-4 | 12.49 | 3.73 | SRS387272 |
|  |  |  | T5-5 | 11.68 | 3.48 | SRS387274 |
|  | A'ba | A'ba Tibetan autonomous prefecture, Sichuan province, China | T6-1 | 11.47 | 3.4 | SRS387276 |
|  |  |  | T6-2 | 18.58 | 5.56 | SRS387278 |
|  |  |  | T6-3 | 14.46 | 4.3 | SRS387280 |
|  |  |  | T6-4 | 18.52 | 5.18 | SRS387282 |
|  |  |  | T6-5 | 15.09 | 4.35 | SRS387284 |
| Wild genus *Sus* and warthog | *Sus barbatus* | Sumatra, Indonesia | 1 | 12.73 | 4.59 | ERS177309 |
|  | *Sus cebifrons* | Philippines | 1 | 19.05 | 6.89 | ERS177341 |
|  | *Sus celebensis* | Sulawesi, Indonesia | 1 | 46.06 | 16.57 | ERS177342 |
|  | *Sus verrucosus* | Java, Indonesia | 1 | 24.04 | 8.79 | ERS177343 |
|  | *Phacochoerus africanus* | Tanzania | 1 | 23.13 | 7.79 | ERS177335 |
