# Supplementary material for: A de novo silencer causes elimination of MITF-M expression and profound hearing loss in pigs
Source: BMC Biol. 2016 Jun 27;14:52. doi: 10.1186/s12915-016-0273-2 (PMC4922063; doi:10.1186/s12915-016-0273-2)
Supplement: Additional file 4: Table S8. — Co-segregated variants detected in re-sequencing and mutation screening. Table S9. Differential expressed genes between MITF R/r and MITF r/r stria vascularis (SVs). Table S10. Expression levels of melanocyte marker genes in porcine SVs. Table S11. Primer pairs used for screening the MITF gene, for qPCR and for mice genotyping. Table S12. Expression levels of SOX family members in porcine SVs. Table S13. Distribution of hearing loss phenotype and genotype in a large Rongchang pig population. (DOCX 55 kb) [file 12915_2016_273_MOESM4_ESM.docx]

## Supplementary Table 8 | Co-segregated variants detected in re-sequencing and mutation screening.

| Position | Variants type | Ref. allele | Mutant allele | Location |
| --- | --- | --- | --- | --- |
| 56176200 | SNP | G | T | Intergenic |
| 56401891 | SNP | C | T | Promoter H |
| 56407324 | SNP | T | C | Promoter H |
| 56461308 | SNP | G | A | Intron 5 |
| 56469862 | SNP | G | C | Intron 4 |
| 56474675 | SNP | G | A | Intron 1 |
| 56475912 | Insertion | A | AT | Promoter M |
| 56477462 | SNP | A | C | Promoter M |
| 56477466-56477470 | Deletion | GGGTA | G | Promoter M |
| 56481335 | Insertion | T | TAC | Promoter M |
| 56482632 | Insertion | T | TTTTAGTTTAAAAAA | Promoter M |
| 56482690 | SNP | C | T | Promoter M |
| 56482691 | SNP | G | T | Promoter M |
| 56482695 | Insertion | C | CAAACTAAGT | Promoter M |
| 56736409 | SNP | A | C | Intergenic |
| 56739845 | SNP | C | A | Intergenic |
| 56749418 | SNP | T | G | Intergenic |
| 56757809 | SNP | T | G | Intergenic |
| 56783824 | SNP | A | G | Intergenic |
| 56787326 | SNP | T | C | Intergenic |
| 56788239 | SNP | C | T | Intergenic |
| 56790522 | SNP | A | C | Intergenic |
| 56824354 | SNP | G | A | Intergenic |
| 56831312 | SNP | C | T | Intergenic |
| 56838861 | SNP | C | T | Intergenic |
| 56850465 | SNP | T | C | Intergenic |

**Supplementary Table 9** | Differential expressed genes between *MITF^R/r^* and *MITF^r/r^* SVs.

| Ensembl Gene ID^a^ | Gene Name | RPKM values of *MITF^R/r^* | RPKM values of *MITF^r/r^* | Log2 Fold change. | p value |
| --- | --- | --- | --- | --- | --- |
| ENSSSCG00000004843 | TRPM1 | 22.68 | 0.01 | -10.63 | 2.65E-18 |
| ENSSSCG00000030767 | TMP-SLA-5 | 17.90 | 0.06 | -8.18 | 1.12E-05 |
| ENSSSCG00000009490 | DCT | 171.48 | 1.19 | -7.15 | 6.28E-171 |
| ENSSSCG00000025038 | KCNJ13 | 11.77 | 0.14 | -6.38 | 1.33E-12 |
| ENSSSCG00000026409 | TYR | 11.76 | 0.18 | -6.05 | 6.94E-06 |
| ENSSSCG00000014923 | RAB38 | 15.53 | 0.36 | -5.43 | 1.64E-06 |
| ENSSSCG00000016821 | SLC45A2 | 27.09 | 0.78 | -5.09 | 9.60E-12 |
| ENSSSCG00000000371 | PMEL | 15.51 | 0.51 | -4.90 | 2.21E-08 |
| ENSSSCG00000012536 | PLP1 | 13.14 | 0.57 | -4.50 | 2.12E-05 |
| ENSSSCG00000009477 | EDNRB | 17.34 | 1.71 | -3.33 | 2.19E-07 |
| ENSSSCG00000006415 | CADM3 | 20.62 | 2.53 | -3.01 | 1.78E-07 |
| ENSSSCG00000025126 | LGI4 | 26.01 | 7.84 | -1.71 | 1.34E-06 |
| ENSSSCG00000001229 | HLA-A | 186.91 | 78.49 | -1.25 | 1.54E-18 |
| ENSSSCG00000000660 | A2M | 167.85 | 70.32 | -1.24 | 2.54E-18 |
| ENSSSCG00000009220 | DMP1 | 59.99 | 27.86 | -1.09 | 1.40E-08 |
| ENSSSCG00000005638 | LCN2 | 105.21 | 51.33 | -1.02 | 1.29E-05 |
| ENSSSCG00000000672 | CLSTN3 | 19.29 | 39.99 | 1.07 | 5.80E-08 |
| ENSSSCG00000030999 | MYH6 | 19.21 | 40.07 | 1.08 | 8.62E-06 |
| ENSSSCG00000024853 | ssc-mir-4332 | 845.70 | 1875.06 | 1.08 | 2.55E-10 |
| ENSSSCG00000017343 | GFAP | 56.16 | 135.93 | 1.29 | 2.55E-23 |
| ENSSSCG00000020149 | 5_8S_rRNA | 164.34 | 475.34 | 1.54 | 6.64E-07 |
| ENSSSCG00000001231 | HLA-B | 15.04 | 53.30 | 1.86 | 4.57E-09 |
| ENSSSCG00000014833 | UCP2 | 9.24 | 33.60 | 1.88 | 8.54E-07 |
| ENSSSCG00000006874 | PALMD | 6.64 | 24.22 | 1.88 | 9.01E-07 |
| ENSSSCG00000006711 | NBPF6 | 5.20 | 19.00 | 1.88 | 9.92E-07 |
| ENSSSCG00000011748 | SLC7A14 | 6.64 | 29.58 | 2.17 | 1.61E-11 |
| ENSSSCG00000030790 | TMP-CH242- 74M17.6 | 11.45 | 114.85 | 3.34 | 5.07E-08 |
| ENSSSCG00000029058 | ADIRF | 0.45 | 93.62 | 7.71 | 5.27E-10 |

^a^ Compare to *MITF^R/r^* SVs, genes down-regulated in *MITF^R/r^* SVs were highlighted by blue color, and those up-regulated were highlighted by red color.

**Supplementary Table 10** | Expression levels of melanocytes marker genes in porcine SVs.

| Ensembl Gene ID | Gene Name | RPKM values of *MITF^R/r^* | RPKM values of *MITF^r/r^* |
| --- | --- | --- | --- |
| ENSSSCG00000008842 | KIT | 43.58 | 44.17 |
| ENSSSCG00000008745 | PROM1 | 2.32 | 1.60 |
| ENSSSCG00000009213 | ABCG2 | 21.39 | 18.96 |
| ENSSSCG00000015377 | ABCB5 | 0.52 | 0.74 |
| ENSSSCG00000017548 | NGFR | 0.74 | 0.63 |
| ENSSSCG00000021155 | POMC | 1.79 | 1.34 |
| ENSSSCG00000012318 | MAGED1 | 289.23 | 287.59 |
| ENSSSCG00000011787 | MAGEF1 | 96.16 | 100.17 |
| ENSSSCG00000012350 | MAGEH1 | 59.38 | 59.24 |
| ENSSSCG00000015116 | MCAM | 14.40 | 14.16 |
| ENSSSCG00000026943 | MRAP2 | 1.59 | 1.22 |
| ENSSSCG00000016175 | MREG | 2.55 | 2.47 |
| ENSSSCG00000013426 | MUM1 | 42.21 | 43.92 |
| ENSSSCG00000012548 | MUM1L1 | 75.52 | 66.90 |
| ENSSSCG00000011033 | VIM | 1939.30 | 2065.14 |
| ENSSSCG00000006612 | S100A10 | 329.87 | 362.11 |
| ENSSSCG00000006610 | S100A11 | 30.56 | 35.42 |
| ENSSSCG00000025269 | S100A11 | 20.59 | 23.28 |
| ENSSSCG00000006589 | S100A12 | 0.58 | 0.38 |
| ENSSSCG00000006582 | S100A14 | 53.99 | 45.48 |
| ENSSSCG00000006581 | S100A16 | 108.24 | 107.66 |
| ENSSSCG00000006580 | S100A2 | 0.34 | 0.63 |
| ENSSSCG00000021084 | S100A6 | 45.59 | 45.47 |
| ENSSSCG00000006590 | S100A8 | 0.49 | 0.27 |
| ENSSSCG00000006588 | S100A9 | 0.40 | 0.22 |
| ENSSSCG00000026140 | S100B | 6090.94 | 6272.46 |
| ENSSSCG00000012147 | S100G | 0.00 | 0.15 |
| ENSSSCG00000022801 | S100PBP | 14.30 | 16.49 |
| ENSSSCG00000006474 | NES | 4.21 | 3.83 |
| ENSSSCG00000002639 | TUBB3 | 0.11 | 0.12 |
| ENSSSCG00000017251 | SOX9 | 83.72 | 74.83 |
| ENSSSCG00000012287 | TFE3 | 34.90 | 32.85 |
| ENSSSCG00000001621 | TFEB | 16.33 | 15.29 |
| ENSSSCG00000016637 | TFEC | 0.44 | 0.30 |
| ENSSSCG00000027420 | KIR1.2 | 20.01 | 5.97 |

**Supplementary Table 11** | Primer pairs used for screening the *MITF* gene, for qPCR and for mice genotyping.

| Usage/Primer pairs | Gene | Location | Oligo sequence 5 ́-3 ́ |
| --- | --- | --- | --- |
| *MITF* mutation screening | | | |
| EA | *MITF* | Exon 1A | F : atggctcactcttgaattctttcctg  R : taataggttttgggctcttcgtgaaa |
| PA | *MITF* | Promoter A  -1.1kb to +0.3kb | F : acagtgcacaatgacctgtt  R : tccctccagctgtcaccact |
| EO-C | *MITF* | Exon 1A  Promoter C  Exon C | F : tacggaggtaccaacaaggaaactgt  R : cgaatcacaacccttcacctactacc |
| EH | *MITF* | Exon 1H | F : aggcacagagaggtgaagtaaattgc  R : ctcagcgctcaacaatctttagtcaa |
| PH | *MITF* | Promoter H  -3.6kb to +0.2kb | F : ctttgggggcagtaaaacatttgata  R : cacagccacagactaacagttccagt |
| EB | *MITF* | Exon 1B | F : ccctcttttccaaagactgagattga  R : cacattgaagagctggcatgtttat |
| EM-4 | *MITF* | Exon M  Intron 1M  Exon 2  Intron 2  Exon 3X  Exon 3  Intron 3  Exon 4 | F : tcaggccaactagaataccaccat  R : tagtgaattggccttgatctgaca |
| PM.1 | *MITF* | Promoter M  -13.5kb to -11.1kb | F : cacatcccaggaagtagttccaaatc  R : attcttcttcctgtgtccactgttcc |
| PM.2 | *MITF* | Promoter M  -11.4kb to -8.1kb | F : aagtaccagccaaagctaagcaactg  R : taccctttggaggtcataccatctgt |
| PM.3 | *MITF* | Promoter M  -8.9kb to -5.9kb | F : tggcttctttcacactatagcagcag  R : tgaacataggctgcaagagagcatag |
| PM.4 | *MITF* | Promoter M  -5.9kb to -2.5kb | F : taaagtggccatcgtactgtctttga  R : tatagtgtgaaagaagccacggacaa |
| PM.5 | *MITF* | Promoter M  -3.0kb to -1.3kb | F : tattctctatttggcactgtcggtga  R : tctctgtgagaatggtgggttcaata |
| PM.6 | *MITF* | Promoter M  -1.8kb to +0.1kb | F : tctcctgggttgtgggctga  R : gcggccccaatccaatgaga |
| E5-6 | *MITF* | Exon 5  Intron 5  Exon 6 | F : gattgccctccagaagtttttctt  R : catgatgggaacgcctactaaaat |
| E7-8 | *MITF* | Exon 7  Intron 7  Exon 8 | F : tcagtgtcagggcttcataaacgat  R : actggttcttctcccatcttcaagg |
| E9 | *MITF* | Exon 9 | F : ttaactaaacacaactggccacca  R : caaatcctctatgcaccacctctt |
| Expression analysis of murine *Mitf* transcript variants | | | |
| Mus-Mitf-A | *Mitf-a* |  | F : gcggatttcgaagtcggggagg  R : ccagccataaacgtcagcgtgc |
| Mus-Mitf-J | *Mitf-j* |  | F : tctcgccgtgtctctgggcatc  R : ccagccataaacgtcagcgtgc |
| Mus-Mitf-H | *Mitf-h* |  | F : gggcttgcagaacaccttaaagg  R : ccagccataaacgtcagcgtgc |
| Mus-Mitf-M | *Mitf-m* |  | F : ggaaatgctagaatacagtcactacc  R : catgcacgacgctcgagagtgc |
| Mus-ACTB | *beta-actin* |  | F : ccccattgaacatggcattg  R : acgaccagaggcatacagg |
| Expression analysis of porcine genes | | | |
| Sus-MITF-A | *MITF-A* |  | F : agagccatgcagtcggaatc  R : gcttatcggaggcttggagg |
| Sus-MITF-H | *MITF-H* |  | F : aagggcttgcagaacacctt  R : gcttatcggaggcttggagg |
| Sus-MITF-M | *MITF-M* |  | F : atagtccaccatttctcattggat  R : gccctgttttgttcttcaaactta |
| Sus-TYR | *TYR* |  | F : aggaatcacaaggcggttatgt  R : tggctctgatacaacaagctgt |
| Sus-DCT | *DCT* |  | F : cattctgctgccaatgatcctg  R : ggagggaagaaaggaaccatgt |
| Sus-KCNJ13 | *KCNJ13* |  | F : gacccagaagaagcactgagaa  R : catccttggtgactatcctccg |
| Sus-TRPM1 | *TRPM1* |  | F : tgccatgaaactgctgacctat  R : ttttcctcatcctcagccttcc |
| Sus-ACTB | *beta-actin* |  | F : cctcttacaagcccacc  R : agtcaggcagcacatcg |
| *Mitf ^mi-ΔM/mi-ΔM^* mouse genotyping | | | |
| Mus-KOtyping | *Mitf* | Exon M | F : ggaaagggacatgcatgcgtcaac  R : acacaatcatgcacacactcccca |
| Rongchang pig genotyping | | | |
| Sus-INTtyping | *MITF* | Promoter M  -7.6kb to -7.4kb | gaaaaccctgcatttgtaggtc  tttccaaaaggtttccaagaat |
| EMSA | | | |
| R probe 1 | *MITF* | Promoter M | ttgaattgacccaagctaggagtgaagataaa |
| r probe 1 | *MITF* | Promoter M | ttgaattgaccaaactaagtcaatttaggagtgaagataaa |
| R probe 2 | *MITF* | Promoter M | atgctaattgttttttagtttaaaaaaa |
| r probe 2 | *MITF* | Promoter M | atgctaattgttttttagtttaaaaaatttagtttaaaaaaa |

**Supplementary Table 12** | Expression levels of SOX family members in porcine SVs.

| Ensembl Gene ID | Gene Name | Chromosome | RPKM values of *MITF^R/r^* | RPKM values of *MITF^r/r^* |
| --- | --- | --- | --- | --- |
| ENSSSCG00000011771 | *SOX2* | 13 | 1.49 | 0.85 |
| ENSSSCG00000022501 | *SOX3* | X | 0.00 | 0.03 |
| ENSSSCG00000001081 | *SOX4* | 7 | 16.26 | 15.16 |
| ENSSSCG00000000567 | *SOX5* | 5 | 6.18 | 5.53 |
| ENSSSCG00000030418 | *SOX6* | 2 | 4.13 | 3.27 |
| ENSSSCG00000023065 | *SOX6-like* | 2 | 1.73 | 1.65 |
| ENSSSCG00000027044 | *SOX7* | 14 | 1.50 | 1.06 |
| ENSSSCG00000017251 | *SOX9* | 12 | 83.72 | 74.83 |
| ENSSSCG00000000115 | *SOX10* | 5 | 0.04 | 0.04 |
| ENSSSCG00000021364 | *SOX10-like* | 5 | 5.20 | 4.50 |
| ENSSSCG00000008649 | *SOX11* | 3 | 0.85 | 0.63 |
| ENSSSCG00000021920 | *SOX12* | 17 | 6.11 | 4.55 |
| ENSSSCG00000015277 | *SOX13* | 9 | 22.37 | 18.40 |
| ENSSSCG00000011656 | *SOX14* | 13 | 0.00 | 0.06 |
| ENSSSCG00000017954 | *SOX15* | 12 | 1.04 | 1.26 |
| ENSSSCG00000006256 | *SOX17* | 4 | 6.31 | 7.50 |
| ENSSSCG00000017051 | *SOX30* | 16 | 0.03 | 0.03 |

**Supplementary Table 13** | Distribution of hearing loss phenotype and genotype in a large Rongchang pig population.

| Band type  Phenotype | 234 bp  *MITF^R/R^* | 234+257 bp  *MITF^R/r^* | 257 bp  *MITF^r/r^* | Other  band type ^a^ | Total |
| --- | --- | --- | --- | --- | --- |
| Normal | 109 | 115 | 0 | 0 | 224 |
| Hearing loss | 0 | 16 | 71 | 0 | 87 |
| Total | 109 | 131 | 71 | 0 | 15.16 |

^a^ Other band type means possible recombined allele between the 14 bp and the 9 bp insertion.
